# The Immunological Profile of Adipose Mesenchymal Stromal/Stem Cells after Cell Expansion and Inflammatory Priming

**DOI:** 10.3390/biom14070852

**Published:** 2024-07-15

**Authors:** Karolien Buyl, Makram Merimi, Robim M. Rodrigues, Saida Rahmani, Mohammad Fayyad-Kazan, Fatima Bouhtit, Noureddine Boukhatem, Tamara Vanhaecke, Hassan Fahmi, Joery De Kock, Mehdi Najar

**Affiliations:** 1 Department of In Vitro Toxicology and Dermato-Cosmetology, Vrije Universiteit Brussel (VUB), 1090 Brussels, Belgium; 2 LBBES Laboratory, Genetics and Immune Cell Therapy Unit, Faculty of Sciences, University Mohammed Premier, Oujda 60000, Moroccobouhtitfatima@gmail.com (F.B.); n.boukhatem@ump.ac.ma (N.B.); 3 Department of Natural and Applied Sciences, College of Arts and Sciences, The American University of Iraq-Baghdad (AUIB), Baghdad 10001, Iraq; 4 Hematology Department, Jules Bordet Institute, Université Libre de Bruxelles, 1000 Brussels, Belgium; 5 Laboratoire d’Hématologie, CHU Mohammed VI, Faculté de Médecine et de Pharmacie d’Oujda, University Mohammed Premier, Oujda 60000, Morocco; 6 Osteoarthritis Research Unit, Department of Medicine, University of Montreal Hospital Research Center (CRCHUM), Montreal, QC H2X 0A9, Canada; 7 Faculty of Medicine, ULB721, Université Libre de Bruxelles, 1070 Brussels, Belgium

**Keywords:** adipose tissue, mesenchymal stromal/stem cells, red blood cell lysis buffer, immune biology, cell passaging, inflammation, Toll-like receptors, cytokines, cell therapy, regenerative medicine

## Abstract

Background: AT-MSCs display great immunoregulatory features, making them potential candidates for cell-based therapy. This study aimed to evaluate the “RBC lysis buffer” isolation protocol and immunological profiling of the so-obtained AT-MSCs. Methods: We established an immune-comparative screening of AT-MSCs throughout in vitro cell expansion (PM, P1, P2, P3, P4) and inflammatory priming regarding the expression of 28 cell-surface markers, 6 cytokines/chemokines, and 10 TLR patterns. Findings: AT-MSCs were highly expandable and sensitive to microenvironment challenges, hereby showing plasticity in distinct expression profiles. Both cell expansion and inflammation differentially modulated the expression profile of CD34, HLA-DR, CD40, CD62L, CD200 and CD155, CD252, CD54, CD58, CD106, CD274 and CD112. Inflammation resulted in a significant increase in the expression of the cytokines *IL-6*, *IL-8*, *IL-1β*, *IL-1Ra*, *CCL5*, and *TNFα*. Depending on the culture conditions, the expression of the TLR pattern was distinctively altered with *TLR1–4*, *TLR7*, and *TLR10* being increased, whereas *TLR6* was downregulated. Protein network and functional enrichment analysis showed that several trophic and immune responses are likely linked to these immunological changes. Conclusions: AT-MSCs may sense and actively respond to tissue challenges by modulating distinct and specific pathways to create an appropriate immuno-reparative environment. These mechanisms need to be further characterized to identify and assess a molecular target that can enhance or impede the therapeutic ability of AT-MSCs, which therefore will help improve the quality, safety, and efficacy of the therapeutic strategy.

## 1. Introduction

The reparative capacity of mesenchymal stromal/stem cells (MSCs) for regenerative purposes is currently a much-investigated research area [1]. Typical MSC features comprise movement to damaged tissue sites, immunotrophic functioning, as well as paracrine signaling, making them highly relevant for clinical applications. The therapeutic effects of MSCs are primarily grounded on their secretome, which includes a diverse panel of biologically functional molecules like chemokines, cytokines, and extracellular vesicles. These intermediaries may alter distinct biological activities like tissue restoration and renewal, cell–progenitor differentiation, and immune or inflammatory reactions [2,3].

MSCs establish a dynamic interplay with the innate and adaptive immune cells during tissue injury. As such, MSCs influence all effectors of the innate and adaptive immune system, showing that their immunomodulatory properties are not HLA-restricted. Moreover, they can alter cell proliferation and other functions of the immune cells involved. Also, the proliferation, cytotoxicity, and IFN-γ production in T lymphocytes and NK cells can be inhibited by MSCs [4]. Lymphocyte proliferation is hindered by blocking the cell cycle at the G0/G1 phase, leading to the induction of apoptotic pathways, impairment of the T cell subset ratio, and inhibition of dendritic cells [5]. Another MSC characteristic is the promotion of macrophage polarization from a pro-inflammatory to an anti-inflammatory phenotype, enhancing tissue regeneration [6]. Distinct MSC features, influencing their properties, can be modified in terms of their microenvironment [7]. Indeed, the migration of MSCs to the injury site and their functions are linked to the local cytokine storm induced by inflammation [8]. MSCs are thus able to sense and control inflammation, highlighting their central role in immune-mediated repair of the damaged and injured tissues [9].

Parameters to consider when isolating MSCs for cellular therapy are the ease of access of the concerned organ/tissue, the number of MSCs that can be obtained, and the harmlessness of the collection technique. Therefore, adipose tissue (AT) seems a prominent organ for MSC isolation due to its universal presence in the human body. Also, large amounts of MSCs can be easily collected with a minimally invasive technique [10,11]. Clinical application of AT-MSCs demands repeated administrations, as well as large numbers of cells, implying extended in vitro cell expansion [12]. However, important changes in the phenotype and morphology, as well as alterations in gene, miRNA, and protein expression levels have been described upon extensive MSC expansion, which eventually leads to cellular senescence [8,13].

From this viewpoint, cell expansion and inflammation can be considered modulators of MSC characteristics and thus should be investigated to empower their clinical value [4]. In this study, we isolated AT-MSCs using the “red blood cell (RBC) lysis buffer” method, which is an easy and robust method to purify MSCs [13,14,15]. We performed an immune-comparative screening of AT-MSCs regarding the expression of 28 cell-surface markers (CD34, CD105, HLA-ABC, HLA-DR, HLA-G1, CD40, CD80, CD86, CD252, CD134, CD29, CD44, CD49, CD54, CD58, CD62L, CD102, CD106, CD146, CD166, CD39, CD73, CD200, CD274, HO-1, CD112, CD155, and ULBP), 6 cytokines/chemokines (*IL-6*, *IL-8*, *IL-1β*, *IL-1Ra*, *CCL5*, and *TNFα*), and 10 Toll-like receptors (*TLR 1–10*) under both parameters cited above. Protein network and functional enrichment analysis were performed to reveal relevant pathways linked to these immunological changes that are associated with trophic and immune regulation. The results of this study can contribute to the unraveling of molecular mechanisms, improving the medicinal capability of AT-MSCs, alongside monitoring the quality, safety, and efficacy of the cellular therapeutic.

## 2. Materials and Methods

### 2.1. AT-MSC Sample Collection and Culture 

Lipoaspirate samples (n = 6) were collected from male and female patients (age range 26–46 [36 ± 9] years) undergoing elective liposuction. The procedure was run in collaboration with the Department of Plastic Surgery of the UZ Brussels (Brussels, Belgium) and the ATLAS clinic (Brussels, Belgium). Informed consent was obtained from all patients involved. This study was approved by the local Ethics Committee of Institut Jules Bordet (CE2387; 9 March 2015) and was performed in accordance with the Declaration of Helsinki. Human AT-MSCs were isolated (RBC lysis protocol) as previously described [14]. Purified AT-MSCs were cultured at a density of 1 × 10^4^ cells/cm^2^ in T75 flasks in Dulbecco’s Modified Eagle Medium (Lonza, Brain-l’Alleud, Belgium) supplemented with 10% (*v*/*v*) fetal bovine serum (FBS) (Hyclone, Perbio Science, Erembodegem, Belgium), 7.33 IU/mL benzyl penicillin (Continental Pharma, Brussels, Belgium), 50 µg/mL streptomycin sulfate (Sigma-Aldrich, Diegem, Belgium), and 2.5 µg/mL fungizone (Invitrogen, Merelbeke, Belgium). Following cultivation and establishment of a small, cryopreserved cell bank, AT-MSCs from primo culture (PM), early passage (P1–2), and late passage (P3–4) were studied.

### 2.2. Inflammatory Priming

Pro-inflammatory stimulation of AT-MSCs was performed as previously described [15]. Briefly, AT-MSCs were exposed for 18 h to a cocktail of cytokines, consisting of 25 ng/mL interleukin (IL)-1β (Peprotech, Rocky Hill, NJ, USA), 50 ng/mL tumor necrosis factor (TNF)-α, 10 ng/mL interferon (IFN)-α, and 50 ng/mL IFN-γ (all from Prospec Inc., Rehovot, Israel).

### 2.3. Flow Cytometry

Flow cytometric analysis was performed as previously described [16], using a MacsQuant analyzer (Miltenyi Biotec, GmbH, Bergisch, Germany) and fluorochrome-labeled monoclonal antibodies (Supplementary Table S1). For 28 markers, both the percentage (%) of positive cells and the mean fluorescence intensity (MFI) were studied. The MFI signifies the amount of each marker per cell that exists within the population.

### 2.4. Transcriptional Profiling

The TLR and cytokine transcriptional profile of AT-MSCs was examined by qPCR as previously described [15]. The gene expression assays applied in this study are cataloged in Supplementary Table S2. All samples were studied in duplicate. Each run comprised two no-template controls, as well as a serial dilution of a pooled cDNA mix from all samples to estimate the qPCR efficiency, hereby using the StepOne Plus System’s Software v2.3. Only data within the PCR efficiency range of 0.85–1.15 were retained. Reference gene expression stability was assessed with the qbasePLUS^®^ software geNorm^®^ v3.4 (Biogazelle, Gent, Belgium). Analyzing a reference gene pool, including glyceraldehyde 3-phosphate dehydrogenase (*GAPDH*), beta-2-microglobulin (*B2M*), hydroxy-methylbilane synthase (*HMBS*), beta-actin (*ACTB*) and ubiquitin C (*UBC*), *GAPDH* showed the highest stability in all analyzed samples and thus was selected as reference gene for normalization of the qPCR data.

### 2.5. Protein–Protein Interaction Network

The protein–protein interaction (PPI) network was constructed with the Search Tool for the Retrieval of Interacting Genes (STRING) online database (http://string-db.org; version 12.0) (accessed on 28 August 2023). The functional annotation and pathway enrichment analysis was also performed using the STRING database.

### 2.6. Statistical Analysis

The flow cytometry data for studying the immunophenotype were statistically analyzed using a two-way ANOVA with Bonferroni’s post hoc test for multiple comparisons. A *p*-value less than or equal to 0.05 is judged as statistically significant (GraphPad Prism 7.00, San Diego, CA, USA). The data are presented as mean ± standard deviation (SD) and originate from 6 AT-MSC donors.

The gene expression data concerning the cytokine and TLR transcriptional profiling were statistically analyzed by applying the unpaired *t*-test. A *p*-value less than or equal to 0.05 is considered statistically significant (GraphPad Prism 7.00, La Jolla, CA, USA). The results are provided as the mean ± standard error of the mean (SEM), originating from 6 donors.

## 3. Results

### 3.1. Morphology 

**Figure 1:**
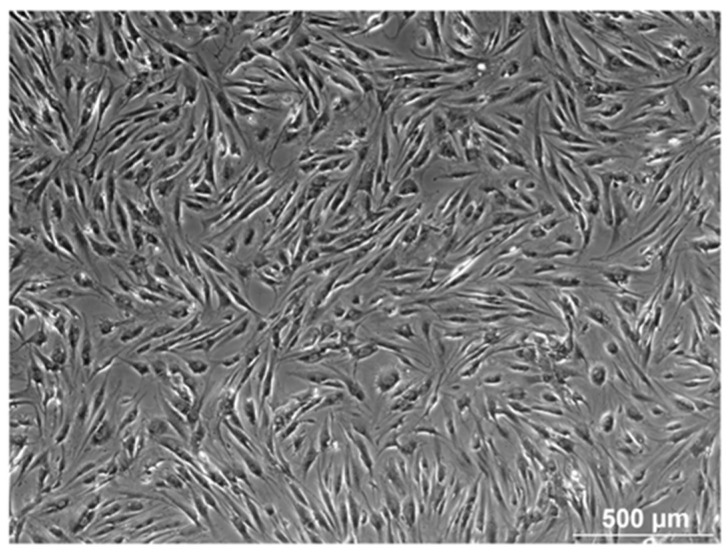
The image is representative of the morphology of AT-MSCs obtained by the RBC lysis buffer protocol and cultured by the classical adherent method. The cells are visualized by light microscopy (scale bar = 500 µm), showing a fibroblast-like cell population.

AT-MSCs were isolated from adipose tissue using the RBC lysis buffer protocol and cultured according to the traditional attachment culture technique. Upon in vitro culture, a heterogeneous population of fibroblast-like cells was observed, showing high adherence to the plastic surface of the culture recipients (Figure 1).

### 3.2. Immune Comparative Screening of AT-MSCs

An immune-comparative screening of AT-MSCs was carried out throughout in vitro cell culture (PM, P1, P2, P3, P4) and inflammatory priming to assess the expression of 28 cell-surface markers involved in various immunological pathway processes. For each investigated marker, the data are presented as the mean ± SEM in Supplementary Table S3 (percentage positive cells) and Supplementary Table S4 (MFI). The tables display the data acquired for each passage (PM-P4) in the standard and the pro-inflammatory settings.

#### 3.2.1. Hematopoietic and Stromal Markers

**Figure 2:**
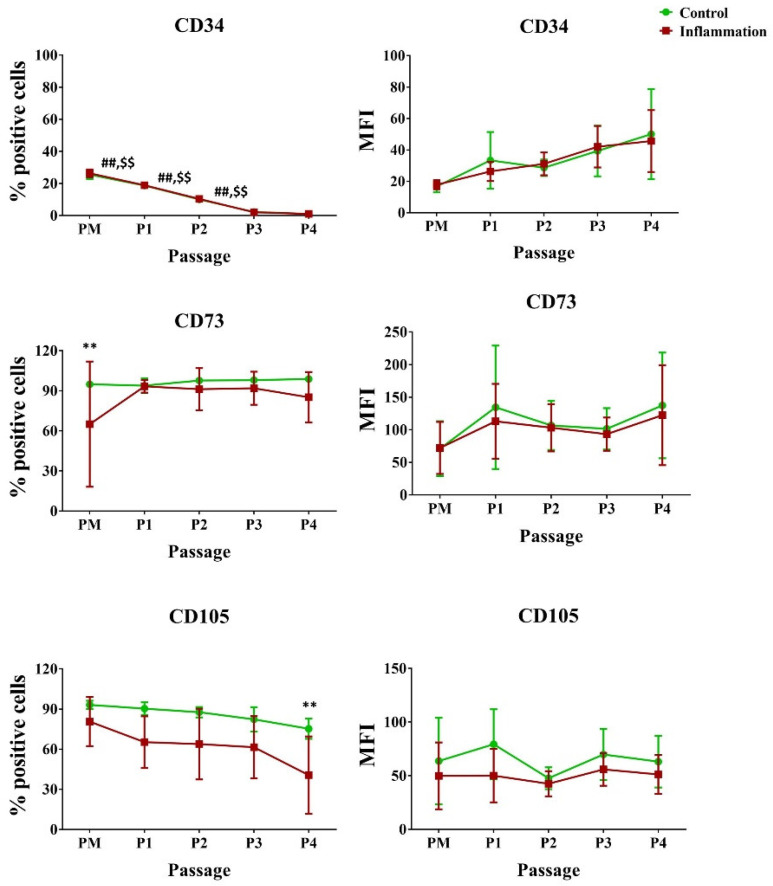
Expression of endothelial/stromal markers in AT-MSCs exposed to inflammatory inducers. The flow cytometry values are expressed as the percentage (%) of positive cells and mean fluorescence intensity (MFI). The values are presented as mean ± SD and originate from six AT-MSC donors. ^##^ Significantly lower percentage or amount per cell in non-stimulated AT-MSCs compared to the consecutive passage (*p*-value < 0.05). ^$$^ Significantly lower percentage or amount per cell in inflammation-stimulated AT-MSCs compared to the consecutive passage (*p*-value < 0.05). ** Significantly lower percentage or amount per cell in inflammation-stimulated AT-MSCs compared to regular AT-MSCs (*p*-value < 0.05).

It was observed that the percentage of AT-MSCs expressing the cluster of differentiation (CD) 34 marker significantly decreased through in vitro expansion, in both the standard and pro-inflammatory setting. Yet, no significant changes could be measured in the amount of CD34 molecules expressed per cell. Likewise, it was seen that inflammatory priming significantly decreased the expression of the stromal markers CD73 and CD105 for the PM (94.83% vs. 65.00%) and P4 (75.33% vs. 40.67%) conditions, respectively. In vitro expansion, on the other hand, had no impact on their expression (Figure 2; Supplementary Tables S3 and S4).

#### 3.2.2. Human Leukocyte Antigens

**Figure 3:**
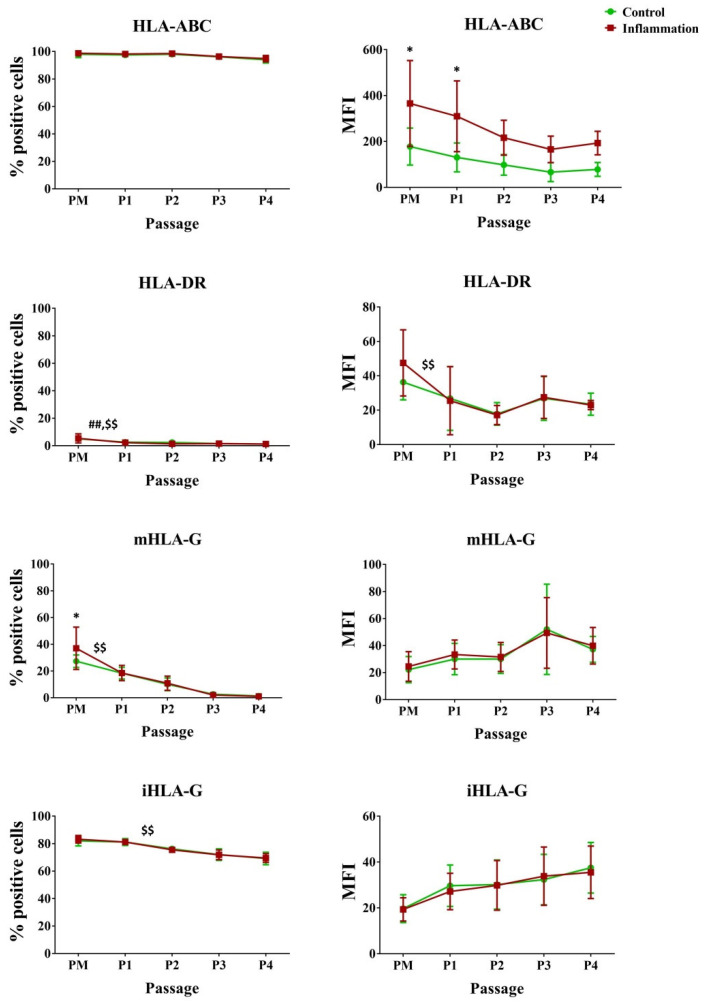
Expression of human leukocyte antigen markers in AT-MSCs exposed to inflammatory inducers. The flow cytometry values are expressed as the percentage (%) of positive cells and mean fluorescence intensity (MFI). The values are presented as mean ± SD and originate from six AT-MSC donors. * Significantly higher percentage or amount per cell in inflammation-stimulated AT-MSCs compared to regular AT-MSCs (*p*-value < 0.05). ^##^ Significantly lower percentage or amount per cell in regular AT-MSCs compared to the consecutive passage (*p*-value < 0.05). ^$$^ Significantly lower percentage or amount per cell in inflammation-stimulated AT-MSCs compared to the consecutive passage (*p*-value < 0.05).

In vitro culture of AT-MSCs significantly decreased HLA-DR expression during the first passage (5.00% vs. 2.50%). The same was found in the pro-inflammatory condition for HLA-DR (PM–P1), intracellular (i) HLA-G (PM-P1), and membrane-bound (m) HLA-G (P1–P2). In addition, for HLA-ABC, inflammatory priming significantly increased the expression of HLA-ABC per cell in AT-MSCs for the PM (177.83 vs. 365.50) and P1 (130.17 vs. 309.67) conditions (Figure 3; Supplementary Tables S3 and S4).

#### 3.2.3. Co-Stimulatory Molecules

**Figure 4:**
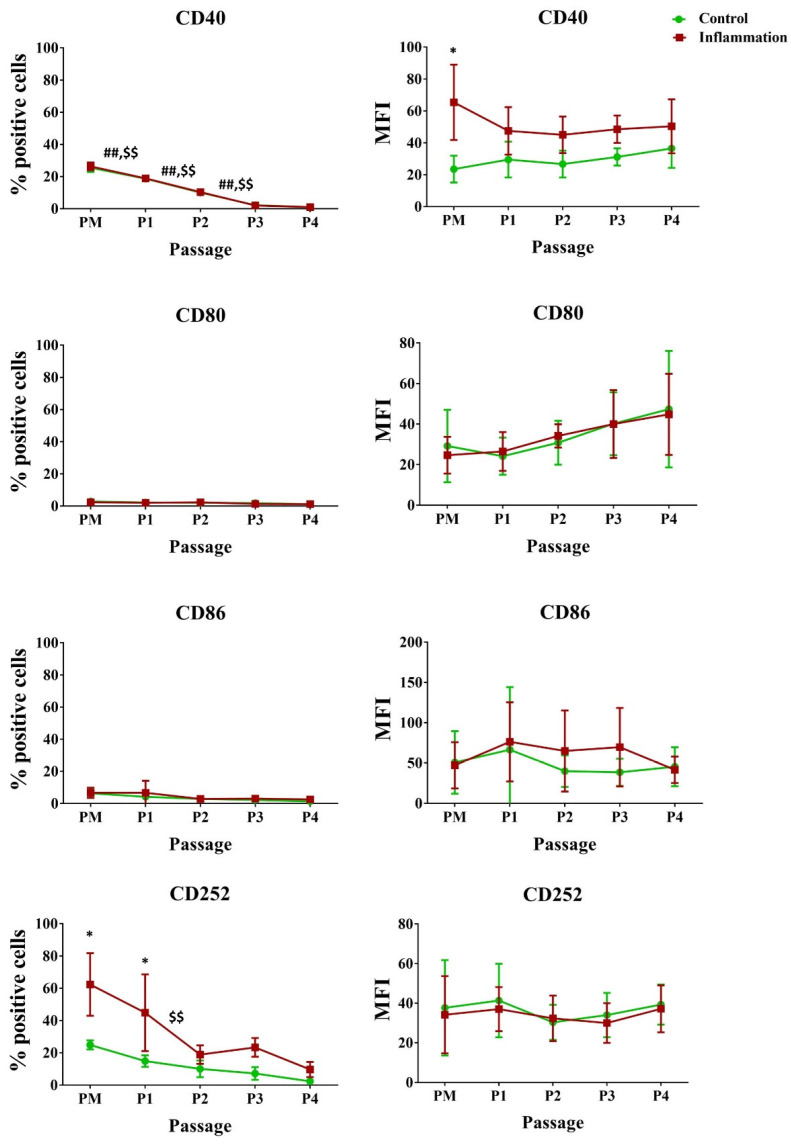
Expression of co-stimulatory molecules in AT-MSCs exposed to inflammatory inducers. The flow cytometry values are expressed as the percentage (%) of positive cells and mean fluorescence intensity (MFI). The values are presented as mean ± SD and originate from six AT-MSC donors. * Significantly higher percentage or amount per cell in inflammation-stimulated AT-MSCs compared to normal AT-MSCs (*p*-value < 0.05). ^##^ Significantly lower percentage or amount per cell in regular AT-MSCs compared to the successive passage (*p*-value < 0.05). ^$$^ Significantly lower percentage or amount per cell in inflammation-stimulated AT-MSCs compared to the successive passage (*p*-value < 0.05).

Under standard culture conditions, only a low to very low percentage of AT-MSCs expressed CD80, CD86, and CD134, while about 25% of AT-MSCs expressed CD40 and CD252. Inflammation particularly influenced the expression of CD252, as a significant upregulation was observed for PM (24.83% vs. 62.33%) and P1 (14.83% vs. 44.83%) upon stimulation of AT-MSCs with a pro-inflammatory cytokine cocktail. In addition, AT-MSC cultivation significantly decreased the CD40 expression, respectively, in normal and pro-inflammatory conditions. Also, during inflammatory priming, for PM, a significant increase was seen for CD40 expressed per cell. No significant changes in expression could be observed for CD80, CD86, and CD134 (Figure 4; Supplementary Tables S3 and S4).

#### 3.2.4. Cell Adhesion Molecules

**Figure 5:**
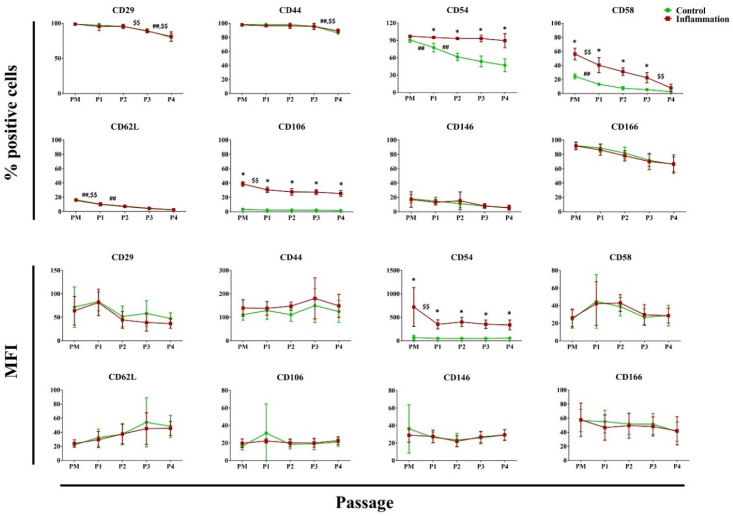
Expression of cell adhesion molecules in AT-MSCs exposed to inflammatory inducers. The flow cytometry values are expressed as the percentage (%) of positive cells and mean fluorescence intensity (MFI). The values are shown as mean ± SD and originate from six distinctive AT-MSC donors. * Significantly higher percentage or amount per cell in inflammation-stimulated AT-MSCs compared to regular AT-MSCs (*p*-value < 0.05). ^##^ Significantly lower percentage or amount per cell in regular AT-MSCs compared to the consecutive passage (*p*-value < 0.05). ^$$^ Significantly lower percentage or amount per cell in inflammation-stimulated AT-MSCs compared to the consecutive passage (*p*-value < 0.05).

In vitro expansion of AT-MSCs in a pro-inflammatory setting versus control leads to a significantly higher percentage of AT-MSCs expressing CD54, CD58, and CD106. This was also observed for CD54 protein expressed per cell. In a standard culture setting, a significant decrease was observed in the percentage of AT-MSCs expressing the cell adhesion molecules CD29, CD44, CD54, CD58, and CD62L during in vitro expansion. Exposure of AT-MSCs to a pro-inflammatory cytokine cocktail demonstrated similar results. As such, the percentage of AT-MSCs expressing CD29, CD44, CD58, CD62L, and CD106 significantly decreased across one or more successive passages during in vitro expansion. No significant changes in expression were observed for CD49e and CD102 (Figure 5; Supplementary Tables S3 and S4).

#### 3.2.5. Immunoregulatory Mediators

**Figure 6:**
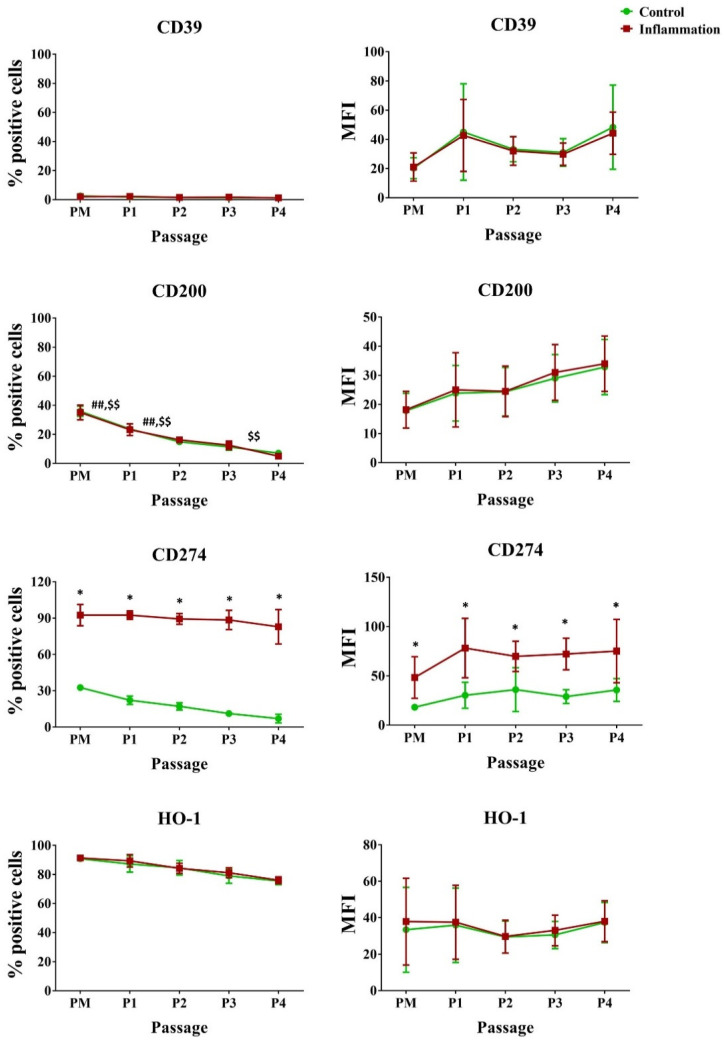
Expression of immunoregulatory molecules in AT-MSCs exposed to inflammatory inducers. Flow cytometric analysis was utilized for the determination of the percentage (%) of positive cells and the protein expression per cell (MFI). The data are expressed as mean ± SD and originate from six AT-MSC donors. * Significantly higher percentage or amount per cell in inflammation-stimulated AT-MSCs compared to regular AT-MSCs (*p*-value < 0.05). ^##^ Significantly lower percentage or amount per cell in regular AT-MSCs compared to the successive passage (*p*-value < 0.05). ^$$^ Significantly lower percentage or amount per cell in inflammatory primed AT-MSCs compared to the consecutive passage (*p*-value < 0.05).

Looking at standard culture settings, a significant percentage of AT-MSCs expressed CD200, CD274, and heme oxygenase (HO)-1. Yet, a significant decrease was observed for CD200 during in vitro cultivation, for both the normal and pro-inflammatory conditions. In contrast, only a very low percentage of AT-MSCs expressed CD39. Inflammation significantly increased the percentage of CD274-expressing cells by approximately 3-fold, representing roughly 90% of the cell population. A significant 2-fold increase was measured for CD274 proteins expressed per cell (Figure 6; Supplementary Tables S3 and S4).

#### 3.2.6. Natural Killer Ligands

**Figure 7:**
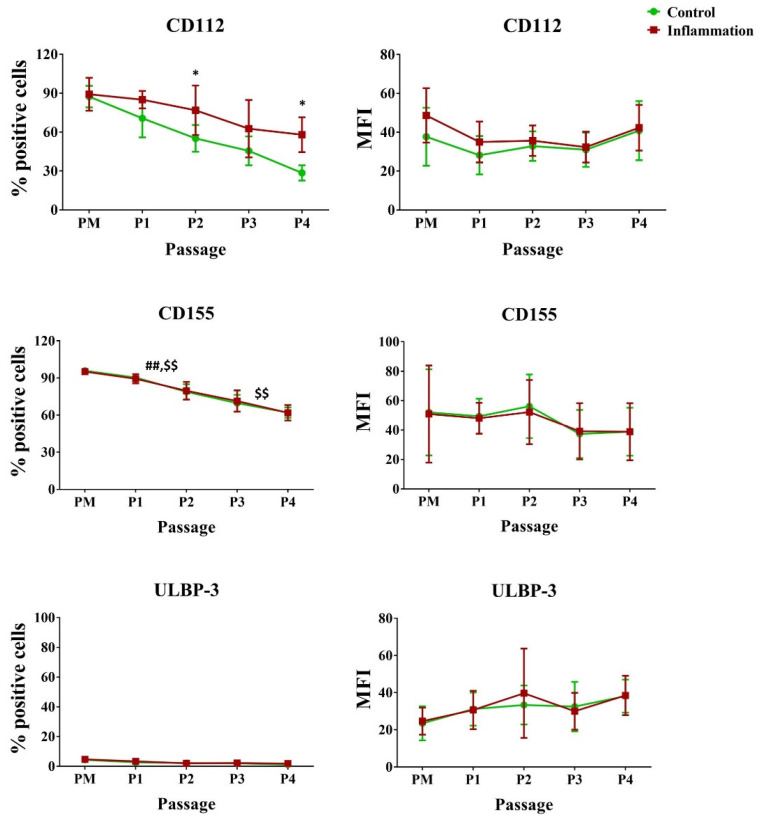
Expression of natural killer ligands in AT-MSCs exposed to inflammatory inducers. Flow cytometric analysis was used to calculate the percentage of positive cells and the protein expression per cell (MFI). The data are presented as mean ± SD and originate from six AT-MSC donors. * Significantly higher percentage or amount per cell in inflammation-stimulated AT-MSCs versus regular AT-MSCs (*p*-value < 0.05). ^##^ Significantly lower percentage or amount per cell in regular AT-MSCs versus the consecutive passage (*p*-value < 0.05). ^$$^ Significantly lower percentage or amount per cell in inflammation-stimulated AT-MSCs versus the consecutive passage (*p*-value < 0.05).

Under normal culture conditions, a high percentage of AT-MSCs in PM constitutively expressed the NK ligands CD112 (87.33%) and CD155 (95.83%). Contrarily, a low percentage (4.33%) of UL16 binding protein 3 (ULBP-3) was measured. In addition, a significant reduction in the percentage of CD155 expressing AT-MSCs was seen throughout in vitro cell culture from P1 (90.33%) to P2 (78.83%). The same is true for P1–P2 and P3–P4 undergoing pro-inflammatory cytokine priming. The percentage of AT-MSCs expressing CD112 at P2 (55.17% vs. 76.83%) and P4 (28.50% vs. 58.00%) was significantly higher in the inflammatory culture conditions. Nonetheless, no significant alterations in protein expression per cell were measured for these markers (Figure 7; Supplementary Tables S3 and S4).

### 3.3. Analysis of the Transcriptional Profile of Cytokines/Chemokines and TLR

A transcriptional profile analysis of distinct cytokines/chemokines and TLR, involved in various immunological pathway processes, was performed in AT-MSCs during cell expansion (early passage (P1–P2) versus late passage (P3–P4)) and inflammatory priming.

#### 3.3.1. Cytokine/Chemokine Expression Pattern

**Figure 8:**
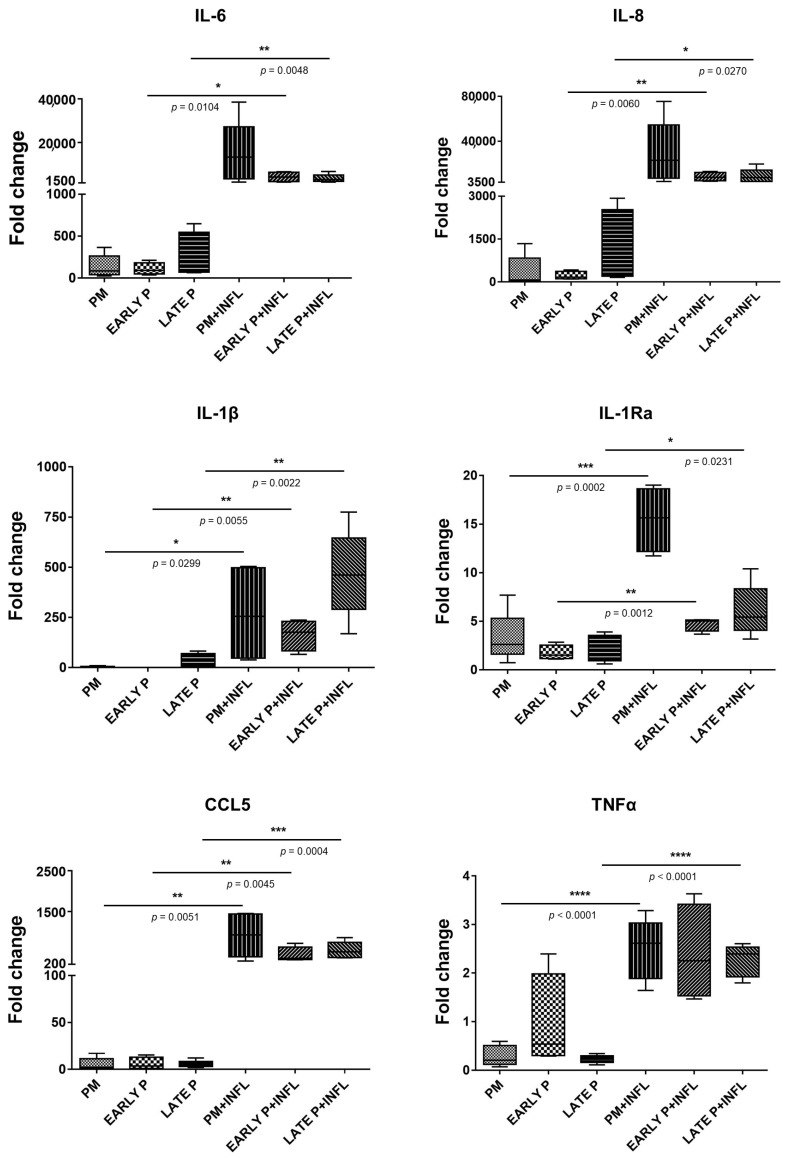
Cytokine and chemokine expression is upregulated in AT-MSCs after inflammatory stimulation. The gene expression levels are determined by qPCR analysis of untriggered and inflamed MSCs (INFL) during the primo culture (PM), early (EARLY), and late (LATE) passage (P). The values are expressed as mean ± SEM compared to the expression of the housekeeping gene (*GAPDH*). *, **, ***, **** Significant increase in expression of inflamed MSCs (INFL) versus regular MSCs (*p*-value: *p* ≤ 0.05, *p* ≤ 0.01, *p* ≤ 0.001, *p* ≤ 0.0001, respectively).

AT-MSCs constitutively expressed a set of cytokines/chemokines at various levels. This expression was differentially modulated, not only by cell expansion, but also by inflammation (Figure 8). Interleukin *(IL)-6* and *IL-8* were expressed at high levels, whereas a lower expression was seen for the C-C motif chemokine ligand *(CCL)5*, *IL-1Ra*, and *IL-1β*. On the contrary, AT-MSCs barely expressed *TNFα*. Independently from the AT-MSC culturing stage, it was observed that all cytokine expression levels were increased in an inflammatory environment. Pro-inflammatory stimulation effectuated that *IL-6*, *IL-8*, *IL-1β*, *IL-1Ra*, *TNFα*, and *CCL5* were secreted at higher levels in contrast to the non-inflammatory situation. In particular, *IL-6* and *IL-8* were significantly upregulated in the early passage (P1–P2) (39.0-fold and 38.9-fold, respectively) and late passage (P3–P4) (13.2-fold and 7.7-fold) conditions. This was also observed for *IL-1β* (343.7-fold for early P and 16.3-fold for late P), *IL-1Ra* (2.7-fold for early P and 2.6-fold for late P), and *CCL5* (74.8-fold for early P and 102.3-fold for late P). In addition, these cytokines/chemokines showed a significantly higher expression in the PM condition (115.8-fold, 4.7-fold, and 166.5-fold, respectively). For *TNFα*, a significantly higher expression was found for PM (8.5-fold) and late P (9.8-fold) after pro-inflammatory stimulation compared to the non-inflammatory setting. As no significant differences were measured in terms of culture period (PM, early P, and late P), it is expected that cell passaging will not influence the cytokine expression profile.

#### 3.3.2. TLR Expression Pattern

**Figure 9:**
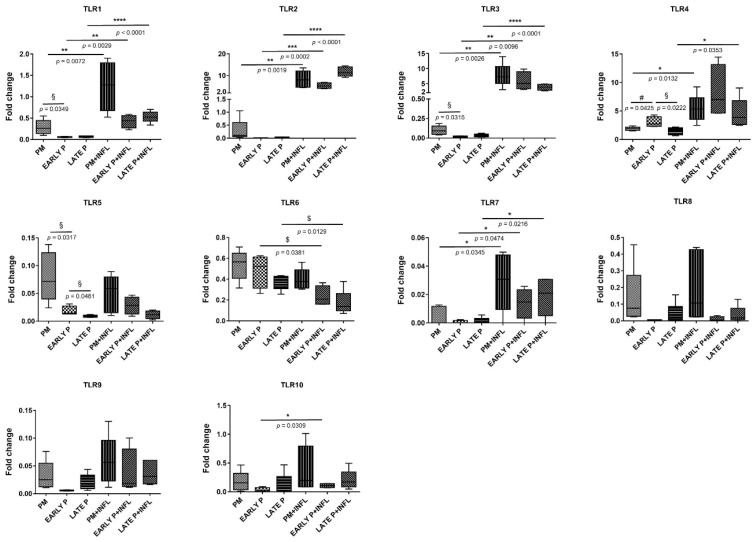
Toll-like receptors (TLRs) are differentially expressed in AT-MSCs after inflammatory stimulation. The gene expression levels are determined by qPCR analysis of untriggered MSCs and inflamed MSCs (INFL) during the primo culture (PM), early (EARLY), and late (LATE) passage (P). The values are expressed as mean ± SEM compared to the expression of the housekeeping gene (*GAPDH*). *, **, ***, **** Significant increase in expression of inflamed MSCs (INFL) versus basic MSCs (*p*-value: *p* ≤ 0.05, *p* ≤ 0.01, *p* ≤ 0.001, *p* ≤ 0.0001, respectively). ^$^ Significant decrease in expression of inflamed MSCs (INFL) versus basic MSCs (*p*-value: *p* ≤ 0.05). ^§^ Significantly higher expression compared to the following passage (*p*-value: *p* ≤ 0.05). ^#^ Significantly lower expression versus the consecutive passage (*p*-value: *p* ≤ 0.05).

AT-MSCs constitutively expressed a set of TLRs at various levels. This expression is differentially modulated, not only by cell expansion, but also by inflammation (Figure 9). As such, *TLR4* showed low expression levels, whereas *TLR1–3* and *TLR5–10* were scarcely identified. After inflammatory stimulation, the expression of *TLR1*, *TLR2*, *TLR3*, *TLR4*, *TLR7*, and *TLR10* was significantly upregulated, whilst that of *TLR6* was significantly downregulated. Notably, a significantly higher expression was observed for *TLR1*, *TLR2*, *TLR3* and *TLR7*, in the PM (4.4-fold, 28.9-fold, 78.6-fold and 6.2-fold, respectively), the early (7.5-fold, 591.6-fold, 309.1-fold and 24.6-fold, respectively), and late (8.2-fold, 494.0-fold, 129.7-fold and 14.9-fold, respectively) passages following pro-inflammatory stimulation versus untreated AT-MSCs. Although *TLR7* expression was hardly measured, a significant upregulation was observed in the inflammatory group. Following inflammatory stimulation, *TLR4* expression was significantly upregulated in the PM (2.8-fold) and late P conditions (3.1-fold). For *TLR10*, a significantly higher expression was observed in the early P condition (3.0-fold) upon inflammation. Alternatively, *TLR6* was significantly lower expressed in the early P (0.5-fold) and late P (0.5-fold) group after treatment with a pro-inflammatory cytokine cocktail. No significant differences were observed for *TLR5*, *TLR8*, and *TLR9* in the control and inflammatory group. Overall, cell passaging tends not to influence TLR expression as no significant differences were observed for the PM, the early P (P1–P2), and the late P (P3–P4) condition, with the exception of *TLR1*, *TLR3*, *TLR4*, and *TLR5*. As such, *TLR1* and *TLR3* show a significantly higher expression in the PM condition versus the early P condition (5.1-fold and 5.3-fold, respectively). The same goes for *TLR5* (4.4-fold) and in addition, a significantly higher expression is observed between the early and late P condition (2.1-fold). The latter is also detected for *TLR4* (2.0-fold), but a significantly lower expression was measured between the PM and early P (0.6-fold).

### 3.4. Protein Interaction Network and Gene Set Enrichment Analysis

**Figure 10:**
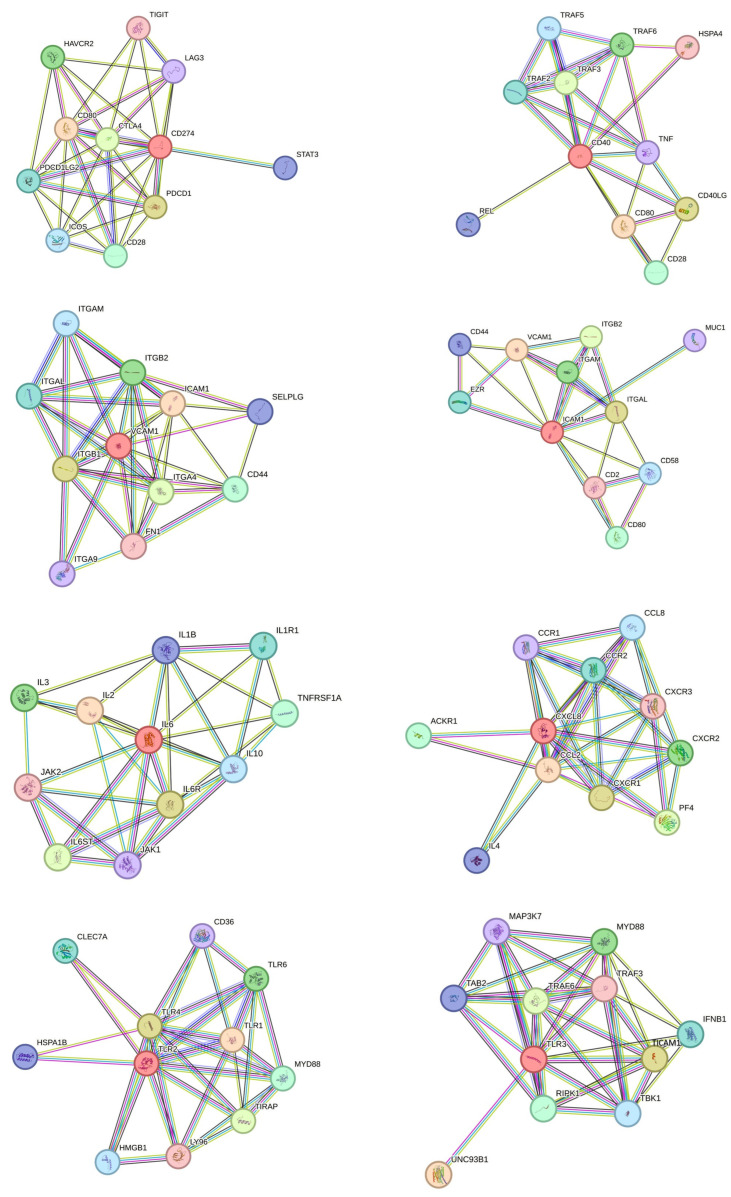
Analysis of known and predicted immunoregulatory molecules, co-stimulatory molecules, cell adhesion molecules, cytokine, and TLR protein interactions using the STRING database. The interactions comprise direct (physical) and indirect (functional) associations. Network nodes represent proteins and edges signify protein–protein associations (specific and meaningful). The lines are considered as the existence of several types of evidence applied in predicting the associations (high confidence score 0.9). The interactions are shown in different colors: green is neighborhood evidence, blue is co-occurrence evidence, purple is experimental evidence, light blue is database evidence and black is co-expression evidence.

Using STRING v12, we visualized protein interaction networks of phenotypic markers (CD274, CD40, CD106/VCAM1, CD54/ICAM1), cytokines/chemokines (IL6, IL8) and TLRs (TLR2, TLR3) (Figure 10) and performed gene set enrichment analysis (Supplementary Figure S1). We observed that several networks, interactions, and associations with different proteins, of which several were investigated in this study, were established. Functional enrichment analysis showed that various biological processes, molecular functions, and cellular components of the investigated proteins were linked to inflammatory and immune responses, showing the relevance of the current study.

## 4. Discussion

Based on their unique biological properties, MSCs are of great interest in the fields of regenerative medicine and immunotherapy [17]. Their therapeutic effects are ascribed to intricate cellular and molecular systems implicated in the regulation and modulation of immune responses. MSCs are recognized as medicinal signaling cells harboring several paracrine signaling molecules and sensing environmental changes, hereby responding by migrating, proliferating, and initiating regenerative mechanisms [18]. Typically, MSCs are considered an attractive cell population for allogeneic stem cell therapy due to their immunomodulatory properties and low immunogenicity [19]. As safety and efficacy are major concerns upon application of cellular therapy, determination of the MSC immunological profile and immunomodulatory properties is key [20]. This information is also indispensable to minimize the risk of host rejection [21]. As inflammation is a key feature of tissue and organ injury, the performance of donor cells should first and foremost be studied within an inflammatory setting [13,22,23]. AT-MSCs are considered a prominent progenitor cell source as they are ubiquitously present in the human body and they can be isolated by a minimally invasive procedure, which is liposuction. As the lipoaspirate is considered as medical waste, no ethical constraints exist upon the use of this stem cell source [10,11,24]. Since cell therapy demands large quantities of donor cells, the need for in vitro cell expansion is a given. Nevertheless, the literature shows that in vitro expansion can alter the MSC phenotype [25]. Therefore, the impact of cell passaging and the environmental setting should be studied before clinical scale-up [26]. In this study, we describe the isolation of AT-MSCs using the RBC lysis buffer protocol, followed by their subsequent immunophenotype characterization, when expanded under standard and inflammatory culture conditions. Further, the impact of cell passaging on the AT-MSC immune profile was studied. To this end, alterations in the AT-MSC immunophenotype were examined by flow cytometry, and the transcriptional characteristics of cytokines/chemokines and TLRs were studied via qPCR.

Flow cytometric analysis revealed that the immunophenotype of AT-MSCs is altered upon inflammation and cell culture. In culture, AT-MSCs displayed a fibroblast-like shape and a high plastic adherence capacity. Although they possess in vitro multilineage differentiation capacity towards osteoblasts and adipocytes, their beneficial effects are most likely attributed to their immunomodulation and trophic ability rather than their multilineage potential [27,28].

We demonstrated that AT-MSCs express the hematopoietic stem cell marker CD34. However, its expression decreases during culture, until its disappearance, also confirmed in our study [10], suggesting that CD34 expression is mainly linked to hematopoietic cells. Nevertheless, CD34-positive non-hematopoietic cell types were described and might indicate a specific subdivision of progenitor cells possessing improved adhesive and homing characteristics [29].

Further, AT-MSCs show a preserved expression of the MSC markers CD73 and CD105 as described by the International Society for Cell and Gene Therapy (ISCT) and are not altered during cell passaging [30]. In previous work, we evaluated the same hematopoietic and stromal markers in AT-MSCs isolated with the Ficoll gradient protocol. For these markers, no differences in expression were observed, showing that both isolation methods preserve the expression of typical MSC markers [31].

Human leukocyte antigens (e.g., HLA-ABC and HLA-DR) and co-stimulatory molecules (e.g., CD40 and CD252) play a central role in adaptive immunity and therefore are the dominant polymorphic proteins targeted in GVHD and allograft rejection [32]. We observed a significant decrease in HLA-DR during in vitro culture of AT-MSCs, indicating that in vitro expanded AT-MSCs are less immunogenic. Previously published flow cytometry studies likewise reported poor to negative expression of HLA-DR on expanded BM-MSCs [33]. In addition, insignificant levels of HLA-DR mRNA were reported for early AT-MSC passages (P2) [12]. HLA-ABC and iHLA-G are ubiquitously expressed during in vitro expansion and inflammatory priming. Inflammation rather than expansion decreases the HLA-G expression in AT-MSCs. On the contrary, it was recently evidenced that HLA-G gene expression significantly decreases throughout passaging AT-MSCs and BM-MSCs [34]. Whereas the examined HLAs show a similar expression pattern as found in Ficoll-isolated AT-MSCs, a twice as large mHLA-G expression was found in freshly (PM) RBC isolated AT-MSCs, suggesting their enhanced immune regulating properties as mHLA-G can inhibit multiple immune cell types [25,31].

The CD40 receptor is a TNF receptor family member and is expressed by B cells, professional antigen-presenting cells, non-immune cells, and tumors. CD40 operates by binding its ligand CD40L, which is transiently expressed on T cells and other non-immune cells under inflammatory circumstances. CD40 is involved in the regulation of a broad spectrum of molecular and cellular processes including the initiation and progression of cellular and humoral adaptive immunity [35]. No significant increase in CD40 expression was measured in our inflammatory-induced AT-MSC cultures, indicating their immune regulatory properties. CD252, also known as OX40L, will activate the TNF receptor OX40 (CD134). The functional activity of effector CD4 and CD8 T cells, NK cells, and NK T cells are clearly regulated by OX40-OX40L interactions. They mediate crosstalk with professional antigen-presenting cells and various cell types for instance mast cells, smooth muscle cells, and endothelial cells. Furthermore, OX40-OX40L interactions modify not only the differentiation, but also the activity of regulatory T cells [36]. As late passage AT-MSCs do not show a significant increase in CD252 expression, they might be preferably used for cell therapy, compared to their early passage counterparts. Pro-inflammatory priming of AT-MSCs did not induce a significant expression of the co-stimulatory molecules CD80, CD86, and CD134, which are necessary for immune response activation. Comparing these findings to Ficoll-isolated AT-MSCs, we can conclude that no differences were observed for CD80 and CD86. The expression of CD40 in RBC-isolated AT-MSCs is approximately 5 times lower in the inflammatory condition than that in Ficoll-isolated AT-MSCs, indicating that RBC-isolated AT-MSCs could be less immunogenic as activation of T-lymphocytes and the immune response will be less prominent compared to that of Ficoll AT-MSCs. Instead, a substantial higher expression was measured for CD252 in the PM and P1 inflammatory conditions of RBC isolated AT-MSCs [31].

Further, we observed that AT-MSCs display a broad panel of cell adhesion molecules (CAMs), assisting in the interaction with activated immune cells and allowing them to react to inflammatory stimuli [26]. As these interactions are indispensable for immunosuppressive functioning, alterations in the CAM expression profile like CD54, CD58, and CD106 could have a significant impact on their immunomodulatory properties. No differences were observed in the expression pattern of CAMs when comparing the Ficoll and RBC isolation protocols [31].

In addition, we found that the expression of CD274, also known as programmed death ligand 1 (PD-L1), is affected by inflammation. The expression of PD-L1 is observed on T and B cells, as well as antigen-presenting cells and in some non-lymphoid tissues. Ligand binding to PD-1 on the surface of T cells moderates immune inhibition. Recent studies described that inhibitory pathways take part in immune evasion by pathogens, indicating that PD-1/PD-L1 is a key mediator in infections. The PD-1/PD-L1 pathway is also crucial in antitumor immune response regulation [37]. As we observed an upregulated expression in inflammatory stimulated AT-MSCs, a high immunosuppressive role is expected. When comparing RBC to Ficoll-isolated AT-MSCs, a similar expression pattern was found for the immunoregulatory molecules (CD39, CD200, CD274, and HO-1) [31].

As part of the innate immune system, NK cells serve as a first-line defense against acute infections and cancer and are involved in the regulation of the adaptive immune response. The activation of NK cells results in cytotoxic degranulation and the production of inflammatory cytokines, eventually leading to the abolishment of target cells [38]. To fulfill this role, NK cells possess activating receptors that target specific NK ligands [39]. Their cytotoxic activity is inhibited by encounter with self-MHC molecules, via inhibitory receptors on their surface that recognize class I HLA molecules [40]. In this work, we observed that AT-MSCs cultured under inflammatory conditions showed an increased expression of the NK ligand CD112, suggesting a higher probability of AT-MSC elimination when used for cell therapy. Possible explanations can be a potential higher NK-mediated cytotoxicity, accompanied by cytokine production. Yet, NK-mediated AT-MSC cytotoxicity can be countered by the upregulated expression of HLA class I molecules (HLA-ABC) [41]. Notwithstanding, the expression pattern of these NK ligands is comparable to the earlier reported AT-MSCs obtained through Ficoll isolation [31]. These findings reveal that the immunophenotype of AT-MSCs is significantly altered upon inflammation and in vitro expansion, which could have a major impact on the immunogenicity and immune-suppressive properties of AT-MSCs, supporting the necessity of our study.

In addition to the immunophenotype, we also evaluated the transcriptional profile of a set of immunomodulatory and trophic mediators like cytokines/chemokines and TLRs. Cytokines modulate or amplify the immune response of cells between innate and adaptive immunity. Chemokines are necessary for immune and inflammatory responses as they are involved in the chemotactic migration of leukocytes. Like this, we distinguish pro- and anti-inflammatory cytokines that encourage or prevent allograft rejection, depending on the environment in which they are secreted. Consequently, it is important to investigate the cytokine/chemokine profile of AT-MSCs under inflammatory culture conditions [42].

We found that AT-MSCs constitutively express the cytokines *IL-6*, *IL-8*, *IL-1 Ra*, *IL-1β*, *TGFβ* and the chemokine *CCL5*. *TNFα*, on the other hand, is barely expressed. Pro-inflammatory stimulation ensures a significantly higher expression of *IL-6*, *IL-8*, *IL-1β*, *IL-1Ra*, *TNFα* and *CCL5*. The expression of *TGFβ*, however, was not altered upon pro-inflammatory stimulation. TGFβ suppresses cytokine production by inhibiting the macrophage and T helper cell (Th) 1 activity and inhibits the expression of IL-6 and TNFα, both pro-inflammatory cytokines. Further, IL-8 and IL-1β promote inflammation, whilst both TNFα and IL-1β will stimulate surface expression of CAMs [43,44]. Since IL-6, TNFα, and IL-1β play a pivotal role in inflammation and graft rejection, anti-cytokine therapies targeting these molecules are being investigated for clinical use. It is known that IL-6 is implied in the suppression of T-cell proliferation and local inflammation. In an inflammatory setting, we demonstrated a significantly higher expression of IL-6, indicating a favorable acceptance of transplanted allogeneic AT-MSCs, hereby enhancing tissue repair and regeneration [45]. Upregulation of the anti-inflammatory cytokine IL-1Ra will also benefit allograft incorporation by blocking IL-1β-mediated cellular alterations by competitive binding on the IL-1β receptor [43]. On the contrary, the pro-inflammatory C-C chemokine ligand, CCL5, also called RANTES, will mediate the recruitment of mononuclear cells, which will destroy the transplanted tissue and, consequently, contribute to transplant dysfunction or loss. Blocking these CCL5 receptors could be a suitable strategy to counteract this process [46]. Lastly, we showed that cytokine expression is not altered upon cell passaging, from which we can assume that there are no major differences in the cytokine profile of young and senescent AT-MSCs. Upon comparison of cytokine expression in Ficoll-isolated AT-MCSs [15], we can assume that for *IL-1β*, a lower expression was seen for both the early and late passage RBC-isolated AT-MSCs when exposed to inflammatory inducers. The same was found for *CCL5*, but in the PM and early P inflammatory conditions. This could indicate that RBC-isolated AT-MSCs are less immunogenic than those obtained through Ficoll gradient centrifugation.

Evaluating the TLR profile of AT-MSCs, we observed that several TLRs were differentially expressed and that this expression is significantly modified upon cell passaging and pro-inflammatory stimulation. TLRs are pattern recognition receptors (PRRs) that play a main role in pathogen-associated molecular pattern (PAMP) recognition and thus the innate immune response. TLR activation triggers intracellular signaling pathways that lead to the induction of inflammatory cytokines and upregulation of co-stimulatory molecules that will activate the adaptive immune response [47]. Many contradictory results exist regarding the role of TLR on the immunomodulatory potential of MSCs. As such, they can modulate MSCs towards an anti- or pro-inflammatory phenotype depending on the ligand concentration, timing, and kinetics of activation [48]. In the case of a pro-inflammatory phenotype, MSCs will be polarized towards an antigen-presenting phenotype, leading to the release of pro-inflammatory cytokines and chemokines, which could facilitate allograft rejection. We showed that *TLR4* is constitutively expressed at low levels and that priming of AT-MSCs ensures a significantly higher *TLR4* expression in the primo culture and late passage condition. Despite the contradictory results in the literature, it is suggested that TLR4 results in the upregulation of pro-inflammatory cytokines like IL-6 and IL-8, which is consistent with our results [47,49]. TLR3, on the other hand, will result in the production of anti-inflammatory molecules. In our study, a significant upregulation of *TLR3* production is observed after inflammatory induction of AT-MSCs, indicating an immunosuppressive effect [47]. Significant upregulation was also seen for *TLR1*, *TLR2*, and *TLR7* after pro-inflammatory stimulation, whereas *TLR6* was significantly downregulated. This is in accordance with what is already described for MSCs in the literature [50]. TLR2 will promote MSC proliferation, impair the capacity to induce the generation of regulatory T-cells, and increase the production of vascular endothelial growth factor (VEGF) [47,51]. Furthermore, TLR2 activation leads to dendritic cell (DC) maturation and secretion of various cytokines involved in immune system activation, like IL-6, IL-8, and TNFα [52]. This is also the case for AT-MSCs in an inflammatory environment. TLR1 will form heterodimers with TLR2 and is critical for the detection of triacylated lipopeptides found in a variety of bacteria and subsequent cytokine production [53]. Regarding the constitutive expression of these TLRs upon AT-MSC passaging, we can conclude that the immune regulatory properties of these cells are enhanced upon passaging. As such, we found that a set (*TLR1*, *TLR3*, *TLR5*) of these TLRs are higher expressed in the primo culture conditions compared to the later passages. Consequently, we believe that immune regulatory properties of AT-MSCs increase by cell passaging. Further, the TLR expression pattern was comparable to that observed in Ficoll AT-MSCs [15]. For most investigated markers, equivalent results were obtained for both isolation protocols. Nonetheless, we observed that the RBC isolation method could offer some advantages concerning AT-MSC immunogenicity (mHLA-G, CD40, IL-1β, CCL5). Taken together with the fact that the RBC isolation protocol is a more robust and straightforward technique to purify AT-MSCs, we propose this technique as the method of choice for the isolation of AT-MSCs in view of cell therapy.

Finally, for a set of immunoregulatory molecules, co-stimulatory molecules, cell adhesion molecules, cytokines, and TLRs, we intended to map the proteins and their functional interactions, as this may provide information on the specificity of individual signaling pathways. Using STRING v12 [54], we visualized protein interaction networks and performed gene set enrichment analysis. We observed that several networks, interactions, and associations with different proteins were established for the phenotypic markers (CD274, CD40, VCAM1, ICAM1), cytokines (IL6; IL8), and TLRs (TLR2, TLR3). Functional enrichment analysis confirmed that several biological processes, molecular functions, and cellular components associated with cytokines and TLRs are implicated in inflammatory and immune responses. The scientific advances in the immune biology of MSCs could be efficiently translated to the clinic if the current limits and gaps can be overcome to achieve tissue regeneration. Cell polarization, differentiation, death, and survival on several immune and tissue cell targets are determining factors to trigger tissue regeneration. The metabolic properties of MSCs in terms of sensing, reacting, and producing metabolites influencing tissue inflammation should be investigated [55]. The inflammatory and aging microenvironment of MSCs may perturb both tissue homeostasis and immunology [56]. The presence of obesity and other challenges may induce a pro-inflammatory state that directly impacts the phenotype, proliferation, and differentiation capacities of adipose-derived stem cells, hereby affecting their regenerative abilities. This could play an essential role in regaining the healthy adipose tissue phenotype and functionality in the context of disorders and diseases by governing the resident pool of MSCs [57]. However, tissue inflammation seems to have no significant influence on in vitro trilineage differentiation potential and proliferative capacity of MSCs in a hepatic injury mouse model [58]. Deciphering the interaction of all these pathways will enable a better understanding of the regulatory network utilized by AT-MSCs for the induction of tissue repair, particularly in the presence of inflammation and a cell-expansion-dependent context. Ultimately, cellular therapy aims to replace damaged resident cells by restoring cellular and molecular environments suitable for tissue repair and regeneration. Growing evidence indicates that some observed therapeutic outcomes of stem cell-based therapy are due to paracrine effects (including extracellular vesicles) rather than long-term engraftment and survival of transplanted cells. Many studies have attempted to exploit the potential of MSCs to differentiate and thus replace damaged resident cells. Currently, no in vivo data demonstrates that MSCs differentiate into complete and functionally resident cells to repair injured tissue [1]. The action of MSCs can be associated primarily with their paracrine properties that reduce the inflammatory response and stimulate the proliferation and differentiation of different local progenitor cells, also known as cell empowerment. MSCs may directly or indirectly favor the generation and proliferation of local progenitors, such as endothelial cells and fibroblasts. The proliferation and functions of keratinocytes, endothelial cells, and fibroblasts are stimulated by molecules present in the secretome of MSCs. Overall, MSCs foster a proregenerative microenvironment that promotes the tissue local repair and regeneration. MSCs effectively participate in the tissue repair process through their immunomodulatory, trophic, antibacterial, antifibrotic, and proangiogenic functions. MSCs also play a central role during the wound-healing process by coordinating between local cells/progenitors and several mediators. A paracrine pathway (secretome) based on the release of different messengers, including regulatory factors, chemokines, cytokines, growth factors, and nucleic acids that can be secreted or packaged into extracellular vesicles, is implicated in the therapeutic properties of MSCs. This secretome coordinates the upregulation of endogenous repair and immunomodulation in the local microenvironment through the crosstalk of MSCs with host tissue cells. Interestingly, the secretome of MSCs was identified as a potential alternative to the cellular product in terms of delivery, safety, and variability of the therapeutic response [59]. The fate and behavior of MSCs may be regulated by different signals (inflammation, tissue damage, infection), which may consequently influence their repair potential. Indeed, MSCs can sense the local stiffness of their microenvironment and adapt their fate to tissue challenges to effectively elicit the proper therapeutic effect. Because the microenvironment is a complex entity, it can be challenging to study and apply. With further research on the changes in their functions, composition, and location in the related pathological process, the mechanism of disease occurrence and development will be clearer, and more innovative treatments based on the use of MSCs will be developed [60]. Such plasticity in functions and phenotype allows MSCs to be used for different therapeutic strategies. In recent years, priming approaches to empower MSCs have been investigated, thereby generating cellular products with improved potential for different clinical applications. Priming with cytokines and growth factors, hypoxia, pharmacological drugs, biomaterials, and different culture conditions, as well as other diverse molecules, are revised for current and future perspectives [61].

Therapeutic applications for MSCs and their derived products often involve in vitro expansion by consecutive passaging, which may significantly alter their therapeutic potential. Understanding how in vitro expansion conditions influence the nature and function of MSCs, and their associated profile, may provide key insights into the underlying mechanisms driving these alterations. Elucidating the dynamic and diverse changes in their immunological profile at each stage of in vitro expansion is a critical next step in the development of standardized, safe, and effective MSC-based therapies.

## 5. Conclusions

In conclusion, RBC-isolated AT-MSCs were highly expandable, sensitive to inflammation, and showed plasticity in their immunological profile. Both cell expansion and inflammatory priming differentially modulated the expression of several markers, such as cytokines/chemokines, and TLR involved in distinct immune, trophic, and inflammatory, responses. Protein network and functional enrichment analysis indicated that these immunological changes allow AT-MSCs to modify their fate to tissue challenges and efficiently obtain the desirable therapeutic outcome. Such plasticity is likely required to generate an immune-reparative setting suitable for tissue repair. Overall, these data can contribute to the knowledge of the molecular mechanisms that might promote or impede the therapeutic capacity of AT-MSCs in a clinical setting and thus to the development of safe, efficient, and high-quality cell therapeutics.

## Data Availability

The data presented in this study might be available depending on the type of demand and the use and are linked to the authorities’ authorization. A request must be sent to the corresponding author and all authors must provide their permission.

## Supplementary Materials

The following supporting information can be downloaded at: https://www.mdpi.com/article/10.3390/biom14070852/s1, Table S1: specifications of the fluorochrome-labeled monoclonal antibodies used for flow cytometric analysis, Table S2: specifications of the TaqMan gene expression assays, Table S3: immunophenotype of AT-MSCs is altered by inflammation and cultivation period, Table S4: the amount of each AT-MSC immunophenotype marker per cell is altered by inflammation and cultivation period. Figure S1: enrichment analysis of phenotypic marker, cytokine, and TLR network interactions and associations.
