# Supplementary material for: The Immunological Profile of Adipose Mesenchymal Stromal/Stem Cells after Cell Expansion and Inflammatory Priming
Source: Biomolecules. 2024 Jul 15;14(7):852. doi: 10.3390/biom14070852 (PMC11275169; doi:10.3390/biom14070852)
Supplement: Supplementary file 1 [file biomolecules-14-00852-s001.zip › biomolecules-3011765-supplementary.pdf]

Supplementary Tables

Supplementary Table S1. Specifications of the fluorochrome labeled monoclonal antibodies used for flow cytometric analysis.

Primary antibody	Order ID	Species	Dilution	Source
**Endothelial and stromal markers**				
anti-CD34-PC5	555823	mouse	1/20	BD
anti-CD105-FITC	326040	mouse	1/20	AC
**Human leukocyte antigens**				
anti-HLA-ABC-PE-Cy5	15998342	mouse	1/20	EB
anti-HLA-DR-PerCP	347402	mouse	1/20	BD
anti-HLA-G1-PE	1P292C100	mouse	1/20	ExBIO
**Immune co-stimulatory molecules**				
anti-CD40-PE	130094135	mouse	1/20	MB
anti-CD80-FITC	11080942	mouse	1/20	EB
anti-CD86-APC	130094876	mouse	1/20	MB
anti-CD252-PE	326308	mouse	1/20	BL
anti-CD134-FITC	350006	mouse	1/20	BL
**Cell adhesion molecules**				
anti-CD29-PE-Cy5	559882	mouse	1/20	BD
anti-CD44-FITC	130095195	mouse	1/20	MB
anti-CD49e-PE	555617	mouse	1/20	BD
anti-CD54-PE	555511	mouse	1/20	BD
anti-CD58-FITC	555920	mouse	1/20	BD
anti-CD62L-Fluorescein	BBA21	mouse	1/20	R&D
anti-CD102-FITC	328507	mouse	1/20	BL
anti-CD106-PE-Cy5	551148	mouse	1/20	BD
anti-CD146-PC5	A22364	mouse	1/20	BC
anti-CD166-PE	559263	mouse	1/20	BD
**Immune regulatory molecules**				
anti-CD39-FITC	328205	mouse	1/20	BL
anti-CD73-PE	344003	mouse	1/20	BD
anti-CD200-APC	329208	mouse	1/20	BL
anti-CD274-PE	557924	mouse	1/20	BD
anti-HO-1-PE	ADI-OSA-111	mouse	1/20	ELS
**Natural killer ligands**				
anti-CD112-PE	337410	mouse	1/20	BL
anti-CD155-PE	337508	mouse	1/20	BL
anti-ULBP-3-PE	FAB1517P	mouse	1/20	R&D

Supplementary Table S2. Specifications of the TaqMan gene expression assays.

Genes	Assay-on-demand ID	Amplicon length (bp)	Supplier
**Housekeeping genes**			
GAPDH	Hs99999905_m1	122	AB
B2M	Hs99999907_m1	75	AB
HMBS	Hs00609296_g1	69	AB
ACTB	Hs99999903_m1	171	AB
UBC	Hs00824723_m1	71	AB
**Cytokines**			
IL-6	Hs00174131_m1	95	AB
IL-8	Hs00174103_m1	101	AB
IL-1b	Hs01555410_m1	91	AB
CCL5	Hs00982282_m1	70	AB
IL-1Ra	Hs00991010_m1	97	AB
TNF-α	Hs00174128_m1	80	AB
**TLR**			
TLR-1	Hs00413978_m1	72	AB
TLR-2	Hs02621280_s1	112	AB
TLR-3	Hs01551079_g1	144	AB
TLR-4	Hs00152939_m1	89	AB
TLR-5	Hs01920773_s1	89	AB
TLR-6	Hs01039989_s1	79	AB
TLR-7	Hs01933259_s1	121	AB
TLR-8	Hs00152972_m1	89	AB
TLR-9	Hs00370913_s1	70	AB
TLR-10	Hs01935337_s1	153	AB

Supplementary Table S3. The expression of 28 cell-surface markers (percentage positive cells) involved in various immunological pathway processes were modulated during *in vitro* cell-expansion (PM, P1, P2, P3, P4) and inflammatory priming as assessed by flow cytometry. Both the constitutive and inflammatory priming results are provided in terms of the culture period. The data are presented as the mean ± SEM of the percentage of AT-MSCs that expressed a given marker from 6 different AT-MSC donors.

**Marker/Culture passage**	**PM**		**P1**		**P2**		**P3**		**P4**	
	Constitutive	*Inflammation*	*Constitutive*	*Inflammation*	*Constitutive*	*Inflammation*	*Constitutive*	*Inflammation*	*Constitutive*	*Inflammation*
Endothelial and stromal markers										
CD34	25,50 ± 1,09	26,33 ± 0,99	18,67 ± 0,80	18,83 ± 0,70	10,00 ± 0,68	10,33 ± 0,62	2,17 ± 0,54	2,00 ± 0,63	1,00 ± 0,00	1,00 ± 0,00
CD73	94,83 ± 0,70	65,00 ± 19,14	93,83 ± 2,23	93,33 ± 1,93	97,67 ± 0,33	91,17 ± 6,46	98,00 ± 0,63	91,83 ± 5,07	98,83 ± 0.17	85,17 ± 7,70
CD105	93,17 ± 1,25	80,67 ± 7,51	90,33 ± 1,94	65,33 ± 7,89	87,67 ± 1,61	63,83 ± 10,79	82,33 ± 3,75	61,50 ± 9,51	75,33 ± 3,10	40,67 ± 11,83
Human leukocyte antigens										
HLA-ABC	98,00 ± 1,00	98,67 ± 0,21	97,50 ± 0,92	98,17 ± 0,40	98,00 ± 0,45	98,50 ± 0,34	96,17 ± 0,48	96,33 ± 0,84	94,00 ± 0,97	94,83 ± 0,95
HLA-DR	5,00 ± 0,78	5,33 ± 1,36	2,50 ± 0,34	2,17 ± 0,60	2,33 ± 0,42	1,33 ± 0,21	1,50 ± 0,22	1,50 ± 0,22	1,17 ± 0,17	1,17 ± 0,17
mHLA-G	27,33 ± 1,91	37,00 ± 6,47	18,33 ± 1,76	18,50 ± 2,32	10,00 ± 1,95	10,83 ± 2,15	2,67 ± 0,56	2,00 ± 0,52	1,33 ± 0,21	1,00 ± 0,00
iHLA-G	82,00 ± 1,53	83,17 ± 1,11	81,17 ± 1,01	81,17 ± 0,75	76,17 ± 0,60	75,50 ± 0,85	72,00 ± 1,71	71,83 ± 1,45	69,17 ± 1,85	69,50 ± 1,31
Co-stimulatory molecules										
CD40	25,50 ± 1,09	26,33 ± 0,99	18,67 ± 0,80	18,83 ± 0,70	10,00 ± 0,68	10,33 ± 0,62	2,17 ± 0,45	2,00 ± 0,63	1,00 ± 0,00	1,00 ± 0,00
CD80	2,83 ± 0,31	2,33 ± 0,42	2,17 ± 0,17	2,00 ± 0,36	2,00 ± 0,00	2,33 ± 0,21	1,83 ± 0,17	1,33 ± 0,21	1,08 ± 0,20	1,17 ± 0,17
CD86	6,33 ± 1,12	6,67 ± 1,31	4,17 ± 0,60	6,67 ± 3,07	2,83 ± 0,31	2,83 ± 0,31	2,17 ± 0,31	3,00 ± 0,63	1,33 ± 0,21	2,50 ± 0,67
CD134	4,00 ± 0,52	3,33 ± 0,62	3,17 ± 0,65	4,33 ± 2,17	2,67 ± 0,42	3,50 ± 0,99	2,67 ± 0,56	3,00 ± 0,63	1,83 ± 0,17	1,50 ± 0,22
CD252	24,83 ± 1,14	62,33 ± 7,92	14,83 ± 1,47	44,83 ± 9,71	10,00 ± 2,11	18,83 ± 2,34	7,17 ± 1,62	23,33 ± 2,38	2,33 ± 0,33	9,67 ± 1,94
Cell adhesion molecules										
CD29	98,83 ± 0.17	98,83 ± 0.17	97,17 ± 1,11	95,50 ± 2,35	95,00 ± 1,18	95,67 ± 1,20	89,33 ± 1,23	88,83 ± 1,08	80,00 ± 2,38	81,17 ± 2,79
CD44	98,17 ± 0,48	97,67 ± 0,56	98,33 ± 0,21	96,50 ± 0,67	98,00 ± 0,36	96,33 ± 1,52	95,67 ± 1,43	95,67 ± 1,78	86,17 ± 0,54	89,00 ± 1,12
CD49e	98,00 ± 0,26	97,17 ± 0,40	97,33 ± 0,49	94,33 ± 1,58	96,67 ± 0,62	95,17 ± 1,08	94,17 ± 1,64	91,83 ± 2,82	90,67 ± 3,18	86,33 ± 4,88
CD54	90,83 ± 1,66	97,17 ± 0,87	77,50 ± 2,99	95,00 ± 0,97	61,50 ± 2,53	93,33 ± 0,33	53,67 ± 3,77	93,33 ± 2,33	47,00 ± 4,52	89,50 ± 4,96
CD58	24,33 ± 1,41	56,17 ± 3,39	13,17 ± 0,79	40,33 ± 4,41	7,50 ± 0,99	31,00 ± 2,25	5,50 ± 0,85	22,50 ± 3,03	2,67 ± 0,56	8,00 ± 2,21
CD62L	15,33 ± 0,67	16,17 ± 0,87	9,83 ± 0,83	10,17 ± 0,95	6,83 ± 0,70	7,33 ± 0,76	4,00 ± 0,58	4,50 ± 0,72	2,33 ± 0,21	2,33 ± 0,21
CD102	3,17 ± 0,40	2,83 ± 0,48	2,17 ± 0,31	12,33 ± 10,74	1,50 ± 0,22	1,33 ± 0,21	1,33 ± 0,21	1,50 ± 0,22	1,00 ± 0,00	1,00 ± 0,00
CD106	3,33 ± 0,76	38,67 ± 1,31	2,33 ± 0,42	30,50 ± 1,56	2,17 ± 0,17	27,67 ± 1,93	2,17 ± 0,17	27,33± 1,38	1,50 ± 0,22	25,33 ± 1,71
CD146	18,17 ± 2,33	17,00 ± 4,42	14,67 ± 2,25	13,17 ± 1,60	11,17 ± 2,01	15,17 ± 5,04	7,83 ± 1,35	8,00 ± 1,21	5,17 ± 0,87	5,67 ± 1,36
CD166	92,17 ± 1,70	91,83 ± 2,21	88,83 ± 2,47	86,17 ± 2,95	81,83 ± 3,34	78,00, ± 2,97	71,67 ± 3,48	70,00 ± 4,68	66,00 ± 4,23	66,50 ± 5,21
Immunoregulatory molecules										
CD39	2,50 ± 0,43	2,00 ± 0,36	1,83 ± 0,48	2,17 ± 0,79	1,50 ± 0,22	1,50 ± 0,22	1,33 ± 0,21	1,67 ± 0,21	1,33 ± 0,21	1,17 ± 0,17
CD200	35,83 ± 1,42	35,00 ± 2,05	23,67 ± 0,80	23,17 ± 1,64	14,83 ± 0,60	16,17 ± 0,60	11,33 ± 0,96	12,50 ± 1,18	7,00 ± 0,52	5,00 ± 0,86
CD274	32,67 ± 1,02	92,50 ± 3,59	22,17 ± 1,40	92,50 ± 1,46	17,17 ± 1,22	89,33 ± 1,80	11,17 ± 0,54	88,50 ± 3,24	7,00 ± 1,48	82,83 ± 5,81
HO-1	90,83 ± 0,75	91,33 ± 0,49	87,17 ± 2,29	89,33 ± 1,78	84,50 ± 2,06	84,17 ± 1,45	79,00 ± 2,08	81,17 ± 1,42	75,50 ± 1,06	76,00 ± 0,93
Natural killer ligands										
CD112	87,33 ± 3,41	89,17 ± 5,18	70,67 ± 6,03	85,00 ± 2,74	55,17 ± 4,21	76,83 ± 7,80	45,50 ± 4,57	62,67 ± 9,06	28,50 ± 2,40	58,00 ± 5,50
CD155	95,83 ± 0,95	95,17 ± 1,11	90,33 ± 1,05	89,33 ± 1,54	78,83 ± 2,50	79,67 ± 2,93	69,67 ± 2,75	71,33 ± 3,51	62,00 ± 1,71	61,83 ± 2,57
ULBP3	4,33 ± 0,62	4,67 ± 0,42	2,67 ± 0,21	3,33 ± 0,76	2,17 ± 0,17	2,00 ± 0,00	2,00 ± 0,00	2,17 ± 0,60	1,17 ± 0,17	1,83 ± 0,31

Supplementary Table S4. The expression of 28 cell-surface markers (mean fluorescence intensity (MFI)) involved in various immunological pathway processes were modulated during *in vitro* cell-expansion (PM, P1, P2, P3, P4) and inflammatory priming as assessed by flow cytometry. Both the constitutive and inflammatory priming results are provided in terms of culture period. The data are presented as the mean ± SEM of the MFI of a given marker expressed by 6 different AT-MSC donors.

**Marker/Culture passage**	**PM**		**P1**		**P2**		**P3**		**P4**	
	Constitutive	*Inflammation*	*Constitutive*	*Inflammation*	*Constitutive*	*Inflammation*	*Constitutive*	*Inflammation*	*Constitutive*	*Inflammation*
Endothelial and stromal markers										
CD34	16,67 ± 1,52	17,83 ± 1,20	33,33 ± 7,36	26,33 ± 2,51	28,67 ± 2,14	31,17 ± 2,96	39,33 ± 6,60	42,00 ± 5,37	50,00 ± 11,69	45,67 ± 8,06
CD73	71,00 ± 17,15	72,17 ± 16,30	134,50 ± 38,71	113,00 ± 23,43	106,67 ± 15,40	103,17 ± 14,80	101,33 ± 13,04	93,33 ± 10,38	137,33 ± 33,11	122,33 ± 31,29
CD105	63,67 ± 16,50	49,83 ± 12,70	79,33 ± 13,31	50,00 ± 10,20	47,67 ± 4,16	42.50 ± 4,81	69,83 ± 9,75	55,83 ± 6,24	63,17 ± 9,89	51,17 ± 7,38
Human leukocyte antigens										
HLA-ABC	177,83 ± 32,91	365,50 ± 76,44	130,17 ± 25,76	309,67 ± 62,91	97,83 ± 18,48	216,17 ± 31,10	66,33 ± 16,85	165,33 ± 23,54	78,17 ± 12,34	192,83 ± 20,94
HLA-DR	36,33 ± 4,20	47,50 ± 7.85	26,83 ± 7,59	25,50 ± 8,09	17,83 ± 2.68	17,17 ± 2,27	26,83 ± 5,20	27,50 ± 5,04	23,50 ± 2.62	23,00 ± 1,10
mHLA-G	22,17 ± 3,94	24,50 ± 4,47	30,00 ± 4,73	33,33 ± 4,38	30,00 ± 4,34	31,50 ± 4,41	52,00 ± 13,63	49,33 ± 10,68	37,17 ± 3,91	39,83 ± 5,53
iHLA-G	19,67 ± 2,49	19,33 ± 2,08	29,67 ± 3,70	27,17 ± 3,24	30,17 ± 4,38	29,83 ± 4,42	32,33 ± 4,50	33,83 ± 5,19	37,50 ± 4,51	35,50 ± 4,68
Co-stimulatory molecules										
CD40	23,50 ± 3,42	65,33 ± 9,66	29,50 ± 4,58	47,50 ± 6,09	26,67 ± 3,44	45,00 ± 4,68	31,17 ± 2,23	48,50 ± 3,50	36,50 ± 5,00	50,33 ± 6,90
CD80	29,17 ± 7,29	24,67 ± 3,71	24,17 ± 3,75	26,50 ± 3,91	30,83 ± 4,44	34,17 ± 2,36	40,17 ± 6,33	40,00 ± 6,84	47,33 ± 11,75	44,83 ± 8,18
CD86	50,67 ± 15,85	47,17 ± 11,69	66,50 ± 31,81	76,33 ± 20,06	39,83 ± 8,04	65,00 ± 20,52	38,50 ± 6,84	69,67 ± 19,87	45,33 ± 9,85	41,50 ± 6,71
CD134	18,67 ± 1,41	18,50 ± 1,15	32,83 ± 3,88	43,83 ± 9,40	34,17 ± 5,19	36,00 ± 4,97	34,00 ± 3,27	33,83 ± 3,37	40,83 ± 4,06	41.67 ± 4.06
CD252	37,67 ± 9,83	34,17 ± 7,96	41,33 ± 7,59	37,00 ± 4,56	30,33 ± 3,62	32,33 ± 4,70	34,00 ± 4,58	30,00 ± 4,10	39,33 ± 4,14	37,17 ± 4,85
Cell adhesion molecules										
CD29	71,67 ± 17,59	64,00 ± 12,47	83,67 ± 8,24	81,67 ± 11,50	51,67 ± 9,06	44,33 ± 7,26	58,33 ± 11,14	38,83 ± 7,52	47,33 ± 4,91	36,67 ± 4,09
CD44	111,00 ± 9,65	139,33 ± 14,64	128,83 ± 15,35	138,00 ± 12,07	111,00 ± 11,49	147,67 ± 7,22	149,67 ± 29,47	180,67 ± 35,72	124,67 ± 18,94	149,00 ± 20,05
CD49e	125,50 ± 22,92	107,00 ± 19,16	95,50 ± 12,67	87,83 ± 16,13	92,50 ± 14,53	81,50 ± 15,97	103,83 ± 10,89	87,50 ± 7,95	75,67 ± 13,29	62,50 ± 12,12
CD54	65,83 ± 20,54	719,50 ± 169,30	49,67 ± 8,46	350,50 ± 40,04	49,00 ± 5,99	399,00 ± 40,45	49,00 ± 5,05	352,00 ± 36,37	55,00 ± 7,19	336,67 ± 42,94
CD58	24,83 ± 4,20	26,00 ± 4,10	44,83 ± 12,42	42,33 ± 10,10	38,83 ± 4,30	43,00 ± 4,24	26,83 ± 3,82	29,67 ± 4,67	28,67 ± 4,74	28,67 ± 3,41
CD62L	22,00 ± 1,16	24,00 ± 2,18	32,50 ± 4,91	29,50 ± 4,68	37,33 ± 6,17	37,50 ± 5,63	54,17 ± 14,29	45,17 ± 9,20	48,50 ± 6,36	45,50 ± 4,10
CD102	25,83 ± 4,61	22,33 ± 1,67	32,83 ± 5,44	34,17 ± 6,36	32,83 ± 4,10	31,17 ± 4,27	35,33 ± 2,96	34,33 ± 4,30	43,00 ± 4,34	40,17 ± 3,76
CD106	16,33 ± 1,61	19,67 ± 1,99	31,33 ± 13,59	22,33 ± 1,02	18,50 ± 2,14	20,17 ± 1,90	19,00 ± 2,68	20,00 ± 1,95	21,17 ± 1,99	22,67 ± 1,86
CD146	36,33 ± 11,27	29,00 ± 3,32	26,50 ± 2,50	27,67 ± 2,94	23,67 ± 3,06	22,00 ± 2,58	26,00 ± 2,78	27,00 ± 2,65	29,17 ± 2,60	29,33 ± 2,44
CD166	56,67 ± 6,52	57,67 ± 9,72	55,17 ± 6,55	46,67 ± 7,32	51,67 ± 6,02	49,33 ± 7,26	51,67 ± 6,15	48,17 ± 5,55	40,83 ± 5,62	42,00 ± 8,13
Immunoregulatory molecules										
CD39	20,17 ± 2,96	21,00 ± 3,97	45,00 ± 13,47	42,67 ± 10,05	33,17 ± 3,49	32,00 ± 4,00	31,00 ± 3,86	29,83 ± 3,14	48,33 ± 11,77	44,17 ± 5,91
CD200	17,83 ± 2,44	18,17 ± 2,57	23,83 ± 3,89	25,00 ± 5,22	24,33 ± 3,40	24,50 ± 3,55	29,00 ± 3,33	31,00 ± 3,91	32,83 ± 3,88	34,00 ± 3,89
CD274	18,17 ± 1,08	48,33 ± 8,59	30,33 ± 5,39	78,17 ± 12,34	36,00 ± 9,10	69,83 ± 6,28	29,00 ± 2,88	72,17 ± 6,54	35,67 ± 4,73	75,17 ± 13,12
HO-1	33,33 ± 9,50	37,83 ± 9,70	35,83 ± 8,31	37,50 ± 8,27	29,33 ± 3,56	29,67 ± 3,66	30,50 ± 3,01	33,00 ± 3,42	37,33 ± 4,46	38,00 ± 4,60
Natural killer ligands										
CD112	37,67 ± 6,09	48,67 ± 5,72	28,17 ± 4,04	35,00 ± 4,30	32,83 ± 3,09	35,67 ± 3,20	31,00 ± 3,62	32,33 ± 3,25	40,83 ± 6,20	42,33 ± 4,81
CD155	52,00 ± 11,94	50,83 ± 13,49	49,33 ± 4,89	48,00 ± 4,28	56,17 ± 8,83	52,17 ± 8,91	37,33 ± 6,69	39,17 ± 7,79	38,83 ± 6,65	38,83 ± 7,92
ULBP3	23,50 ± 3,75	24,67 ± 2,97	31,17 ± 3,65	30,67 ± 4,22	33,33 ± 4,29	39,67 ± 9,80	32,50 ± 5,44	30,00 ± 4,05	38,17 ± 3,64	38,50 ± 4,33

Supplementary Figures

Supplementary Figure S1. Functional enrichments and statistics of phenotypic marker (CD274, CD40, VCAM1, ICAM1), cytokine (IL6, IL8), and TLR (TLR2, TLR3) network interactions and associations. Gene Ontology (GO) is used to implement enrichment analysis for biological processes, molecular functions, and cellular components.

CD274

Network Stats

number of nodes:	11	expected number of edges:	10
number of edges:	35	PPI enrichment p-value:	1.2e-09
average node degree:	6.36		
avg. local clustering coefficient:	0.851		

Functional enrichments in your network

Note: some enrichments may be expected here (why?)

explain columns

Biological Process (Gene Ontology)

GO-term	description	count in network	strength	false discovery rate
GO:0002652	Regulation of tolerance induction dependent upon immune r...	2 of 3	3.08	0.00074
GO:0045590	Negative regulation of regulatory T cell differentiation	2 of 5	2.86	0.0014
GO:0002643	Regulation of tolerance induction	3 of 20	2.43	7.00e-05
GO:0046007	Negative regulation of activated T cell proliferation	2 of 15	2.38	0.0074
GO:0070234	Positive regulation of T cell apoptotic process	2 of 16	2.35	0.0081

(more ...)

Cellular Component (Gene Ontology)

GO-term	description	count in network	strength	false discovery rate
GO:0098636	Protein complex involved in cell adhesion	3 of 52	2.01	0.0011
GO:0009897	External side of plasma membrane	7 of 388	1.51	3.88e-07
GO:0009986	Cell surface	9 of 894	1.26	8.87e-08
GO:0005886	Plasma membrane	11 of 5544	0.55	0.00045
GO:0016021	Integral component of membrane	10 of 5670	0.5	0.0093

CD40

Network Stats

number of nodes:	11	expected number of edges:	11
number of edges:	25	PPI enrichment p-value:	0.000171
average node degree:	4.55		
avg. local clustering coefficient:	0.824		

Functional enrichments in your network

Note: some enrichments may be expected here (why?)

explain columns

Biological Process (Gene Ontology)

GO-term	description	count in network	strength	false discovery rate
GO:0097400	interleukin-17-mediated signaling pathway	3 of 9	2.78	1.20e-05
GO:1903721	Positive regulation of I-kappaB phosphorylation	2 of 7	2.71	0.0012
GO:0035666	TRIF-dependent toll-like receptor signaling pathway	2 of 7	2.71	0.0012
GO:0048304	Positive regulation of isotype switching to IgG isotypes	2 of 10	2.55	0.0021
GO:0023035	CD40 signaling pathway	2 of 10	2.55	0.0021

(more ...)

Molecular Function (Gene Ontology)

GO-term	description	count in network	strength	false discovery rate
GO:0005174	CD40 receptor binding	2 of 2	3.25	0.0012
GO:0031996	Thioesterase binding	4 of 11	2.81	1.17e-07
GO:0005164	Tumor necrosis factor receptor binding	6 of 32	2.53	7.68e-11
GO:0031435	Mitogen-activated protein kinase kinase kinase binding	2 of 16	2.35	0.0236
GO:0031625	Ubiquitin protein ligase binding	5 of 299	1.48	0.00036

(more ...)

Cellular Component (Gene Ontology)

GO-term	description	count in network	strength	false discovery rate
GO:0035631	CD40 receptor complex	5 of 12	2.87	2.35e-10
GO:0009898	Cytoplasmic side of plasma membrane	4 of 174	1.61	0.00054
GO:0098552	Side of membrane	9 of 611	1.42	1.52e-09
GO:0009897	External side of plasma membrane	5 of 388	1.36	0.00044
GO:0005887	Integral component of plasma membrane	8 of 1706	0.92	0.00021

CD106 – VCAM1

Network Stats

number of nodes:	11	expected number of edges:	11
number of edges:	37	PPI enrichment p-value:	3.15e-10
average node degree:	6.73		
avg. local clustering coefficient:	0.806		

Functional enrichments in your network

Note: some enrichments may be expected here (why?)

explain columns

Biological Process (Gene Ontology)

GO-term	description	count in network	strength	false discovery rate
GO:0140039	Cell-cell adhesion in response to extracellular stimulus	2 of 2	3.25	0.0011
GO:0045963	Negative regulation of dopamine metabolic process	2 of 2	3.25	0.0011
GO:2000363	Positive regulation of prostaglandin-E synthase activity	2 of 3	3.08	0.0013
GO:0043315	Positive regulation of neutrophil degranulation	2 of 4	2.95	0.0016
GO:0007161	Calcium-independent cell-matrix adhesion	2 of 4	2.95	0.0016

(more ...)

Molecular Function (Gene Ontology)

GO-term	description	count in network	strength	false discovery rate
GO:0030369	ICAM-3 receptor activity	2 of 3	3.08	0.0028
GO:0001851	Complement component C3b binding	2 of 11	2.51	0.0121
GO:0001968	Fibronectin binding	2 of 30	2.08	0.0490
GO:0005178	Integrin binding	9 of 159	2.01	5.11e-14
GO:0005518	Collagen binding	3 of 66	1.91	0.0055

(more ...)

Cellular Component (Gene Ontology)

GO-term	description	count in network	strength	false discovery rate
GO:0034688	Integrin alphaM-beta2 complex	2 of 2	3.25	0.00017
GO:0034687	Integrin alphaL-beta2 complex	2 of 2	3.25	0.00017
GO:0034668	Integrin alpha4-beta1 complex	2 of 3	3.08	0.00023
GO:0008305	Integrin complex	6 of 30	2.55	2.25e-11
GO:0035579	Specific granule membrane	3 of 90	1.78	0.0012

CD54 – ICAM1

Network Stats

number of nodes:	11	expected number of edges:	10
number of edges:	24	PPI enrichment p-value:	0.000227
average node degree:	4.36		
avg. local clustering coefficient:	0.834		

Functional enrichments in your network

Note: some enrichments may be expected here (why?)

explain columns

Biological Process (Gene Ontology)

GO-term	description	count in network	strength	false discovery rate
GO:0045963	Negative regulation of dopamine metabolic process	2 of 2	3.25	0.0016
GO:2000363	Positive regulation of prostaglandin-E synthase activity	2 of 3	3.08	0.0022
GO:0022614	Membrane to membrane docking	3 of 5	3.03	1.89e-05
GO:0043315	Positive regulation of neutrophil degranulation	2 of 4	2.95	0.0026
GO:0002291	T cell activation via T cell receptor contact with antigen bou...	2 of 4	2.95	0.0026

(more ...)

Molecular Function (Gene Ontology)

GO-term	description	count in network	strength	false discovery rate
GO:0030369	ICAM-3 receptor activity	2 of 3	3.08	0.0035
GO:0001851	Complement component C3b binding	2 of 11	2.51	0.0181
GO:0005178	Integrin binding	5 of 159	1.75	8.21e-05
GO:0050839	Cell adhesion molecule binding	6 of 560	1.28	0.00055
GO:0044877	Protein-containing complex binding	7 of 1261	1.0	0.0019

(more ...)

Cellular Component (Gene Ontology)

GO-term	description	count in network	strength	false discovery rate
GO:0034688	Integrin alphaM-beta2 complex	2 of 2	3.25	0.00021
GO:0034687	Integrin alphaL-beta2 complex	2 of 2	3.25	0.00021
GO:0008305	Integrin complex	3 of 30	2.25	0.00010
GO:0098636	Protein complex involved in cell adhesion	4 of 52	2.14	7.78e-06
GO:0001772	Immunological synapse	2 of 43	1.92	0.0138

(more ...)

TLR2

Network Stats

number of nodes:	11	expected number of edges:	10
number of edges:	31	PPI enrichment p-value:	1.42e-07
average node degree:	5.64		
avg. local clustering coefficient:	0.843		

Functional enrichments in your network

Note: some enrichments may be expected here (why?)

explain columns

Biological Process (Gene Ontology)

GO-term	description	count in network	strength	false discovery rate
GO:0070340	Detection of bacterial lipopeptide	3 of 3	3.25	4.77e-07
GO:1903974	Positive regulation of cellular response to macrophage colo...	2 of 2	3.25	0.00016
GO:0042496	Detection of diacyl bacterial lipopeptide	2 of 2	3.25	0.00016
GO:0042495	Detection of triacyl bacterial lipopeptide	2 of 2	3.25	0.00016
GO:0038124	Toll-like receptor TLR6:TLR2 signaling pathway	2 of 2	3.25	0.00016

(more ...)

Molecular Function (Gene Ontology)

GO-term	description	count in network	strength	false discovery rate
GO:0035663	Toll-like receptor 2 binding	3 of 3	3.25	1.59e-06
GO:0001875	Lipopolysaccharide immune receptor activity	3 of 5	3.03	3.24e-06
GO:0035325	Toll-like receptor binding	7 of 12	3.02	3.57e-16
GO:0035662	Toll-like receptor 4 binding	2 of 4	2.95	0.0011
GO:0071723	Lipopeptide binding	3 of 10	2.73	1.52e-05

(more ...)

Cellular Component (Gene Ontology)

GO-term	description	count in network	strength	false discovery rate
GO:0035355	Toll-like receptor 2-Toll-like receptor 6 protein complex	2 of 2	3.25	0.00035
GO:0035354	Toll-like receptor 1-Toll-like receptor 2 protein complex	2 of 2	3.25	0.00035
GO:0046696	Lipopolysaccharide receptor complex	2 of 5	2.86	0.00087
GO:0030670	Phagocytic vesicle membrane	3 of 76	1.85	0.0014
GO:0045335	Phagocytic vesicle	4 of 139	1.71	0.00034

(more ...)

Reference publications (PubMed)

publication	(year) title	count in network	strength	false discovery rate
PMID:30131804	(2018) Toll-Interleukin 1 Receptor Domain-Containing Adapt...	5 of 5	3.25	1.54e-10
PMID:25784622	(2015) Association of TLR1, TLR2, TLR4, TLR6, and TIRAP p...	5 of 5	3.25	1.54e-10
PMID:24848367	(2014) Cross-sectional analysis of Toll-like receptor variants...	5 of 5	3.25	1.54e-10
PMID:23255565	(2013) Racial variation in toll-like receptor variants among w...	5 of 5	3.25	1.54e-10
PMID:22238472	(2012) Variants in toll-like receptor 1 and 4 genes are associ...	5 of 5	3.25	1.54e-10

TLR3

Network Stats

number of nodes:	11	expected number of edges:	11
number of edges:	36	PPI enrichment p-value:	7.06e-10
average node degree:	6.55		
avg. local clustering coefficient:	0.784		

Functional enrichments in your network

Note: some enrichments may be expected here (why?)

explain columns

Biological Process (Gene Ontology)

GO-term	description	count in network	strength	false discovery rate
GO:0008063	Toll signaling pathway	2 of 3	3.08	0.00041
GO:0035666	TRIF-dependent toll-like receptor signaling pathway	2 of 7	2.71	0.0012
GO:0002756	MyD88-independent toll-like receptor signaling pathway	3 of 12	2.65	1.61e-05
GO:0097527	Necroptotic signaling pathway	2 of 8	2.65	0.0015
GO:0034138	Toll-like receptor 3 signaling pathway	2 of 9	2.6	0.0017

(more ...)

Molecular Function (Gene Ontology)

GO-term	description	count in network	strength	false discovery rate
GO:0031996	Thioesterase binding	2 of 11	2.51	0.0217
GO:0035325	Toll-like receptor binding	2 of 12	2.47	0.0217
GO:0005123	Death receptor binding	2 of 21	2.23	0.0439
GO:0032813	Tumor necrosis factor receptor superfamily binding	4 of 50	2.16	8.07e-05
GO:0005126	Cytokine receptor binding	5 of 270	1.52	0.00054

(more ...)

Cellular Component (Gene Ontology)

GO-term	description	count in network	strength	false discovery rate
GO:0097342	Ripoptosome	2 of 6	2.78	0.0020
GO:0035631	CD40 receptor complex	2 of 12	2.47	0.0053
GO:0010008	Endosome membrane	7 of 540	1.37	3.74e-06
GO:0005768	Endosome	9 of 1030	1.19	3.11e-07
GO:0098588	Bounding membrane of organelle	8 of 2125	0.83	0.00066

IL6

Network Stats

number of nodes:	11
number of edges:	33
average node degree:	6
avg. local clustering coefficient:	0.748
expected number of edges:	11
PPI enrichment p-value:	5.01e-08

Functional enrichments in your network

Note: some enrichments may be expected here (why?)

explain columns

Biological Process (Gene Ontology)

GO-term	description	count in network	strength	false discovery rate
GO:0002384	Hepatic immune response	2 of 2	3.25	0.00019
GO:1900100	Positive regulation of plasma cell differentiation	2 of 3	3.08	0.00030
GO:0010573	Vascular endothelial growth factor production	3 of 7	2.89	4.34e-06
GO:0070102	interleukin-6-mediated signaling pathway	5 of 14	2.81	1.13e-09
GO:1901731	Positive regulation of platelet aggregation	4 of 12	2.78	7.47e-08

(more ...)

Molecular Function (Gene Ontology)

GO-term	description	count in network	strength	false discovery rate
GO:0019970	interleukin-11 binding	2 of 3	3.08	0.0020
GO:0004921	interleukin-11 receptor activity	2 of 3	3.08	0.0020
GO:0005131	Growth hormone receptor binding	2 of 11	2.51	0.0078
GO:0070851	Growth factor receptor binding	7 of 135	1.97	1.11e-09
GO:0004896	Cytokine receptor activity	3 of 96	1.75	0.0075

(more ...)

Cellular Component (Gene Ontology)

GO-term	description	count in network	strength	false discovery rate
GO:0005896	interleukin-6 receptor complex	3 of 3	3.25	5.29e-06
GO:0070110	Ciliary neurotrophic factor receptor complex	2 of 3	3.08	0.0019
GO:0098802	Plasma membrane signaling receptor complex	4 of 194	1.57	0.0019
GO:0043235	Receptor complex	5 of 418	1.33	0.0019
GO:0005887	Integral component of plasma membrane	6 of 1706	0.8	0.0392

(more ...)

IL8

Network Stats

number of nodes:	11
number of edges:	33
average node degree:	6
avg. local clustering coefficient:	0.807
expected number of edges:	10
PPI enrichment p-value:	1.72e-08

Functional enrichments in your network

Note: some enrichments may be expected here (why?)

explain columns

Biological Process (Gene Ontology)

GO-term	description	count in network	strength	false discovery rate
GO:1904783	Positive regulation of NMDA glutamate receptor activity	2 of 6	2.78	0.0017
GO:0002407	Dendritic cell chemotaxis	4 of 18	2.6	1.87e-07
GO:0070098	Chemokine-mediated signaling pathway	10 of 82	2.34	5.09e-19
GO:0048245	Eosinophil chemotaxis	2 of 17	2.32	0.0079
GO:0071624	Positive regulation of granulocyte chemotaxis	3 of 28	2.28	0.00015

(more ...)

Molecular Function (Gene Ontology)

GO-term	description	count in network	strength	false discovery rate
GO:0004918	interleukin-8 receptor activity	2 of 2	3.25	0.00047
GO:0019959	interleukin-8 binding	2 of 3	3.08	0.00066
GO:0019958	C-X-C chemokine binding	3 of 6	2.95	4.74e-06
GO:0016494	C-X-C chemokine receptor activity	3 of 8	2.83	7.00e-06
GO:0031727	CCR2 chemokine receptor binding	2 of 6	2.78	0.0016

(more ...)

Cellular Component (Gene Ontology)

GO-term	description	count in network	strength	false discovery rate
GO:0009897	External side of plasma membrane	5 of 388	1.36	0.0026
